# T-cell diversity and exclusion of blood-derived T-cells in the tumor microenvironment of classical Hodgkin Lymphoma

**DOI:** 10.1038/s41375-024-02490-6

**Published:** 2024-12-17

**Authors:** Nicole Seifert, Sarah Reinke, Johanna Grund, Berit Müller-Meinhard, Julia Richter, Thorsten Heilmann, Hans Schlößer, Michaela Kotrova, Monika Brüggemann, Peter Borchmann, Paul J. Bröckelmann, Michael Altenbuchinger, Wolfram Klapper

**Affiliations:** 1 Department of Medical Bioinformatics, University Medical Center Göttingen, Göttingen, Germany; 2 Department of Pathology, Hematopathology Section, University Hospital Schleswig-Holstein Campus, Kiel, Germany; 3 Franziskus-Hospital Harderberg, Osnabrück, Germany; 4 Center of Molecular Medicine, Cologne Translational Immunology, University of Cologne, Cologne, Germany; 5 Department of Hematology and Oncology, University Hospital Schleswig-Holstein Campus, Kiel, Germany; 6 Department I of Internal Medicine, Centre for Integrated Oncology Aachen Bonn Cologne Düsseldorf (CIO ABCD), Faculty of Medicine and University Hospital of Cologne, University of Cologne, Cologne, Germany; 7 German Hodgkin Study Group (GHSG), Cologne, Germany; 8 Max-Planck Institute for Biology of Ageing, Cologne, Germany

**Keywords:** Immunopathogenesis, Lymphoma

## Abstract

The Tumor Microenvironment (TME) in classical Hodgkin Lymphoma (HL) contains abundant immune cells and only few neoplastic Hodgkin and Reed-Sternberg cells (HRSC). We analyzed the T-cell receptor (TCR) repertoire to detect T-cell expansion in the TME and blood. In contrast to solid cancer tissue, T-cells in the TME of HL are highly polyclonal at first diagnosis and show only minor clonal expansion during anti-PD1 immune checkpoint blockade (ICB). At relapse and during ICB, pre-amplified T-cell populations increase in the TME of solid cancers but to a much lesser extent in HL. In contrast, T-cell populations in the peripheral blood of HL patients display higher clonality than healthy controls reaching clonality levels comparable to solid cancer. However, pre-amplified blood T-cells in HL patients show only minor additional clonal expansion during ICB. Moreover, blood-derived T-cells do not repopulate the TME of HL to the same extent as observed in solid cancers. Thus, the T-cell repertoire in the TME of HL appears unique by a relatively low clonal T-cell content and the exclusion of clonally expanded T-cells from the peripheral blood. Exclusion of clonally expanded tumor-specific T-cells from the TME may present a novel mechanism of immune evasion in HL.

## Introduction

Classical Hodgkin Lymphoma (HL) is characterized by an abundant non-neoplastic tumor microenvironment (TME) predominantly composed of T-cells [1]. Neoplastic Hodgkin and Reed-Sternberg cells (HRSC) comprise only 3–5% of cells in the tumor lesions [1]. HRSC secrete abundant cytokines and seem to actively shape the T-cell-rich TME [2]. So far it is unclear whether these T-cells represent actively attracted unspecific cells forming a protective TME or a continuously ongoing specific and clonally expanded anti-lymphoma immune response. Clinically, immune checkpoint blockade (ICB) with anti-PD1 antibodies is highly effective in HL and increasingly explored in the first-line setting [3–6].

The rapid clinical responses [7] as well as molecular data gathered from sequential tissues biopsies during anti-PD1 first-line treatment suggest a mode of action different to solid tumors [8]. In the peripheral blood of HL patients, T-cell-receptor (TCR) repertoire analysis suggested CD4 + T-cell expansion to be associated with response to ICB, raising the question whether an immune response by CD4 + T-helper cells is occurring and boosted by ICB [9]. A deeper understanding of the immune response and the mode of action of ICB in HL will be crucial to further optimize therapeutic approaches.

To gain insight into the T-cell response in HL, we analyzed TCR repertoires to evaluate T-cell expansion in blood and tissue specimens of patients with HL at baseline, relapse after conventional chemotherapy, or early during ICB-based first-line treatment. We illustrate differences in T-cell dynamics in blood and TME between HL and solid cancers suggesting that an anti-lymphoma T-cell response occurs primarily outside the TME in HL. Importantly, these externally expanded T-cells appear to not efficiently enter and/or expand within the HL TME.

## Methods

TCR beta chain CDR3 regions repertoires were sequenced and processed by service of Adaptive Biotechnologies (Seattle, Washington) according to the providers specifications. Only productive TCR rearrangements were further processed as follows. For each sample, CDR3 sequences with the same amino acid clonotype were aggregated to clones using the sum of their counts.

HD12/15 TCR repertoires were sequenced by Amplicon TRBVJ next-generation sequencing of lymphoma tissue as described previously [10], filtered for productive rearrangements, and analyzed with the ARResT/Interrogate immunoprofiler (arrest.tools/interrogate) [11] and a subgroup analyzed with a previosuly published method [10]. (Supplementary Table 1).

The cohorts analyzed herein are outlined in Supplementary Table 1 for tissue and Supplementary Table 2 for blood specimen. Basic characterization of the specimen in respect to sequences analyzed are outlined in Supplementary Table 3. Overall *n* = 734 specimen and 77.989.945 sequences were analyzed.

CD4+ and CD8+ cells PBMC samples were sorted using a FACS Aria III Fusion (BD Bioscience). For cell sorting the PBMC samples were stained with FITC anti-human CD3 (clone SK7), PE anti-human CD4 (clone SK3) and Pacific Blue anti-human CD8 (clone SK1) antibodies (BioLegend) followed by a live/dead staining with Live/Dead Far Red (ThermoFisher) according to the manufacturer (5 µl antibody per 1 × 10^6^ cell, 1 µl Live/Dead stain per 1 × 10^6^ cell).

Five parameters were used to describe TCR repertoires in order to study diversity/clonality and dynamics: Simpson’s Clonality (SC), Percentage of Singletons (PoS), clonal expansion, clone overlap, and antigen distribution. SC is a measure for clone homogeneity with higher values (higher clonality) indicating lower diversity of a given sample. SC was calculated for each sample with

$${SC}=\,\sqrt{{\sum }_{i=1}^{n}{f}_{i}^{2}}$$

where *n* is the total number of different clones and *f*_*i*_ is the productive frequency of clone *i*. PoS represents the proportion of clones that are detected only once (Singletons) in a given sample. Clonal expansion was calculated as previously described [9], but with one variation. If more than one follow-up sample was available, clonal expansion was calculated from the initial to the last available follow-up timepoint per patient. The proportion of Expanded Singletons (ES, count ≤1 at baseline) and Expanded Non-Singletons (ENS, count >1 at baseline) were calculated and visualized separately. Clone overlap describes the proportion of clones in one TCR repertoire of a patient (sample 1) that can also be detected in a second TCR repertoire of the same patient (sample 2). Clone overlap was calculated for each patient with ${CO}=\,{\sum }_{i=1}^{m}{f}_{i}$, where *m* is the total number of different clones in sample 1 that can also be found in sample 2 and *f*_*i*_ is the productive frequency of clone *i*.

If not indicated otherwise, statistical testing was performed using Wilcoxon rank-sum test, or, for paired samples of the same patient (e.g., at different time points), using paired *t*-test.

Details regarding antigen distribution and dynamics are summarized in the Supplementary Methods.

DNA and RNA extraction was performed using different commercial kits (Qiagen, AmpTec, or Thermo Fisher Scientific) according to the manufacturer’s instructions. Gene expression analysis was performed using the NanoString PanCancer Immune Profiling Panel as previously described [8]. Background thresholding and normalization were performed by the NSolver software (version 4.0; NanoString Technologies). The R package “nanostring” was used to perform quality controls [12]. Samples with ≤50% of genes detected above the limit of detection were removed (*n* = 2). Gene expression counts of the remaining samples (*n* = 93) were log 2‐transformed and subsequently centered around the sample mean.

Immunofluorescence multistaining (immunohistochemistry; IHC) and whole slide image (WSI) analysis were performed as previously described [13].

Univariate linear regression and Pearson correlation were used to analyze the correlation between SC and the cell surface markers in the TME assessed by IHC. A multivariate linear regression model was used to analyze also the association between SC in the TME (response variable) and several clinical patient characteristics (predictor variables), which included the patient’s age, sex, clinical stage, remission status after receiving 4x Nivolumab, presence of B symptoms, and allocation to study arm A or B in the NIVAHL trial.

Survival times of the HD12/15 cohort were calculated as previously described [1, 14]. Cox proportional hazards regression models were calculated for overall survival (OS) and progression-free survival (PFS), where SC served as predictor variable.

## Results

**Fig. 1:**
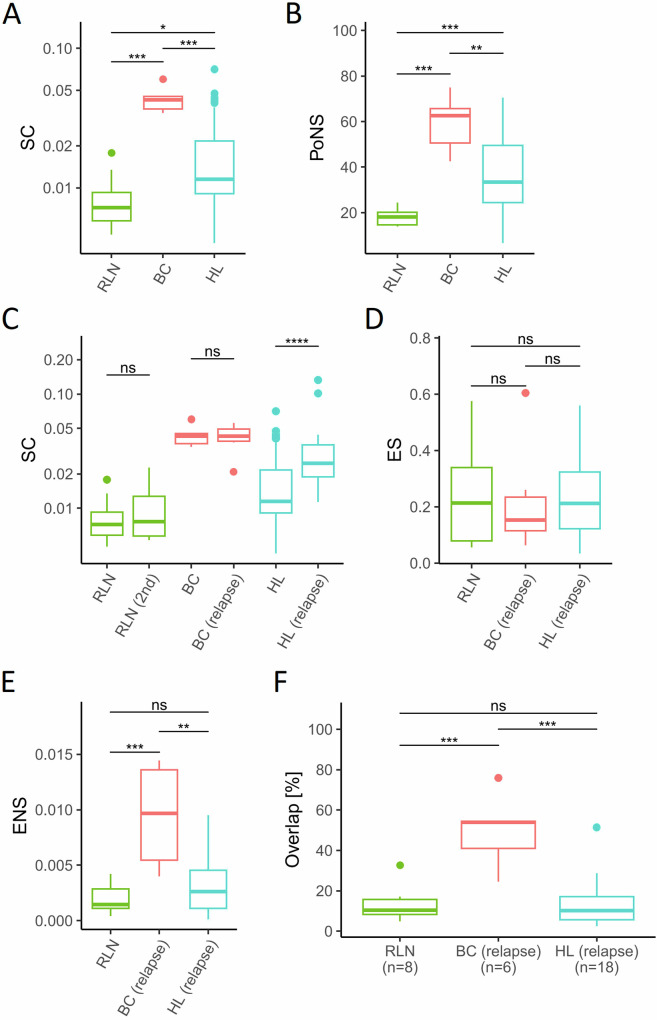
T-cell receptor (TCR) repertoire analysis in healthy tissue and in tumor microenvironments before and at relapse. **A** Simpson’s Clonality (SC) in reactive lymph nodes (RLN, *n* = 8), treatment-naïve breast cancer (BC, *n* = 6), treatment-naïve Hodgkin Lymphoma (HL, *n* = 108). **B** Percentage of Non-Singletons (PoNS) in RLN, BC, and HL as shown in (**A**). **C** SC in paired biopsies of patients with RLN as shown in (**A**) and a subsequent biopsy (RLN 2nd, *n* = 8), of treatment-naïve BC and BC at relapse after surgery (*n* = 6), of treatment-naïve HL and HL at relapse (subset of HL as paired biopsies at relapse after chemotherapy, *n* = 18). **D** Clonal expansion of singletons (ES) between two biopsies of RLN, BC, and HL pairs shown in (**B**). **E** Clonal expansion of non-singletons (ENS) between two biopsies of RLN, BC, and HL pairs shown in (**B**). **F** Overlap of TCR sequences as percentage of TCR sequences with the same amino acid sequence in the treatment naive biopsy which were also detected in a follow-up biopsy of the same patient. Box and whiskers plots with median indicated as horizontal bar. ns not significant (*p* > 0.05), *p 0.05–0.01, **p < 0.01, ***p < 0.0001, ****p < 0.00001.

Clonal expansion of T-cells is a feature that has been observed in solid cancers after ICB [15]. In line with this interpretation, bulk analysis of localized treatment naive breast cancer (BC) specimens showed a higher clonality (median SC = 0.0429) compared to reactive lymph nodes (RLN; median SC = 0.0072) which served as a control (*p* = 0.0007, Fig. 1A). In contrast, the TME of treatment naive HL displayed a rather polyclonal TCR pattern. Clonality in HL biopsies (median SC = 0.0115) was only slightly higher than in reactive lymphoid tissue and significantly lower compared to BC (Fig. 1A, *p* = 0.014 and *p* = 0.0003, respectively). The content of unique single-copy TCR sequences (singletons) showed a distribution inverse to clonality. Polyclonal specimen, such as RLN and HL, displayed the highest PoS (Supplementary Fig. 1A) Of note, a specimen with a low SC value can be considered polyclonal in its entirety but may still contain clonal T-cell populations. In fact, the content of TCR sequences detected at least twice in the (percentage of non-singletons) is significantly higher in HL compared to reactive lymphoid tissue albeit lower than in solid cancer (Fig. 1B).

Thus, T-cells in the TME of treatment-naive HL are highly diverse and clonal expansion seems limited by bulk TCR analysis. Clonally expanded T-cells, i.e., presumably lymphoma-specific T-cells, are thus present only as small populations which might be locally prevented from expansion in the TME of HL. To distinguish these two possibilities, we first searched for TCR sequences of known specificity against Epstein-Barr-Virus (EBV) and correlated their presence with the detection of EBV in HRSC detected by LMP1 immunohistochemistry or EBER in situ hybridization [8]. We did not detect an enrichment of TCR sequences annotated to be EBV-specific in HL with EBV-positive versus EBV-negative HRSC in two independent cohorts of treatment naive HL comparing 12 EBV+ versus 73 EBV− cases and 11 EBV+ versus 43 EBV− cases, respectively (Supplementary Figs. 2 and 3). Unfortunately, public databases are limited in the number of TCR sequences annotated for EBV proteins which are expected in HRSC (EBNA1, LMP1, LMP2a) and the number or TCR sequences obtained in our analysis varies (Supplementary Table 3). Thus, our analysis suffers from low sensitivity. Nevertheless, we consider the absence or at least low frequency of EBV-specific TCR sequences in EBV-positive HL in combination with the polyclonality of TCR repertoires to support the hypothesis of a TME mostly devoid of specific T-cells directed against the neoplastic cells in HL.

Next, we analyzed TCR dynamics between multiple biopsies of the same patients to identify expansion of TCR as a surrogate for an anti-lymphoma immune reaction of T-cells. First, we analyzed bulk TCR clonality in pairs of primary and relapse samples of the same patient. We consider these episodes of clinical re-occurrence as a model of re-exposure of the immune system to the same tumor. Two consecutive biopsies of RLN (RLN and RLN 2nd) in the same patient served as control. We did not detect an increase of clonality in BC specimens between primary and relapse biopsies probably because maximum levels of clonality have already been reached at first presentation (Fig. 1C). However, HL specimens displayed higher TCR clonality at relapse after conventional chemotherapy (Fig. 1B, *p* < 0.0001). Again, the PoS was inversely proportional to clonality (Supplementary Fig. 1B). The finding in HL suggests that HL prevents clonal expansion in the treatment-naïve TME but clonal expansion is gradable at relapse. Of note, even at relapse, TCR clonality in HL was lower compared to treatment naive BC specimen (*p* = 0.047, Fig. 1C, Supplementary Table 4) indicating that even at re-occurrence an accumulation of clonally expanded T-cells is hampered in the HL TME.

To better understand TCR dynamics, we analyzed subsets of TCR sequences defined by their frequency in the first biopsy. To this end, TCR sequences detected in the primary biopsy were separated into single-copy (singletons) and multiple-copy TCR sequences (non-singletons), the latter reflecting pre-amplified clones [9]. The expansion of singletons and non-singletons was studied comparing the primary and relapse biopsy as defined in the Methods Section. Expansion of singletons and non-singletons in RLN (comparing a first and the subsequent biopsy of the same patient) was assessed to define random TCR dynamics in lymphoid tissue at multiple time points (Fig. 1D, E). As expected, based on the definition of singletons, the level of expansion is higher in singletons than in non-singletons. In paired biopsies of HL, expanded singletons and non-singletons were not significantly different than in reactive lymphoid tissue. In contrast, BC specimens displayed an expansion of non-singletons at relapse that was higher than in reactive tissue and HL (Fig. 1D, E, *p* = 0.001 and *p* = 0.004, respectively). These data show that in BC pre-amplified TCR clones (non-singletons) expand at relapse in the TME—a finding compatible with the scenario of a re-exposure of a primed immune system to the antigenic repertoire of this solid cancer. In contrast, clonal expansion of pre-amplified T-cell clones appears to be effectively prevented in primary, treatment-naive HL as well as at relapse. This phenomenon may be explained by (i) ineffective immune priming in treatment-naive HL (anergy) (ii) blockade of clonal expansion of lymphoma-specific T-cells despite their presence in the TME (inhibition) or (iii) prevention of infiltration by primed, clonally pre-expanded T-cells (exclusion) at relapse. Of note, our analysis evaluates bulk tumor/TME. We have no access to single-cell data which may allow detection of small T-cell clones that escape bulk TCR analysis [16]. Nevertheless, our findings suggest that the increase in clonality of relapsed HL is not linked to expansion of preexisting clones. To further evaluate this hypothesis, we analyzed the TCR clone overlap in the treatment-naïve TME with the TME at relapse. Reactive lymph nodes with first and sequential biopsy from the same patient served as a negative control. The percentage of clones in the treatment-naïve TME that were also detected at relapse was significantly higher in BC than in HL and RLN (Fig. 1F, *p* = 0.0002 and *p* = 0.001, respectively), whereas we observed no significant differences between HL and RLN. These findings are in line with low level of clonal expansion in the TME of HL compared to BC and support the hypothesis that the observed increased clonality in HL at relapse (Fig. 1B) is not linked to expansion of preexisting clones.

**Fig. 2:**
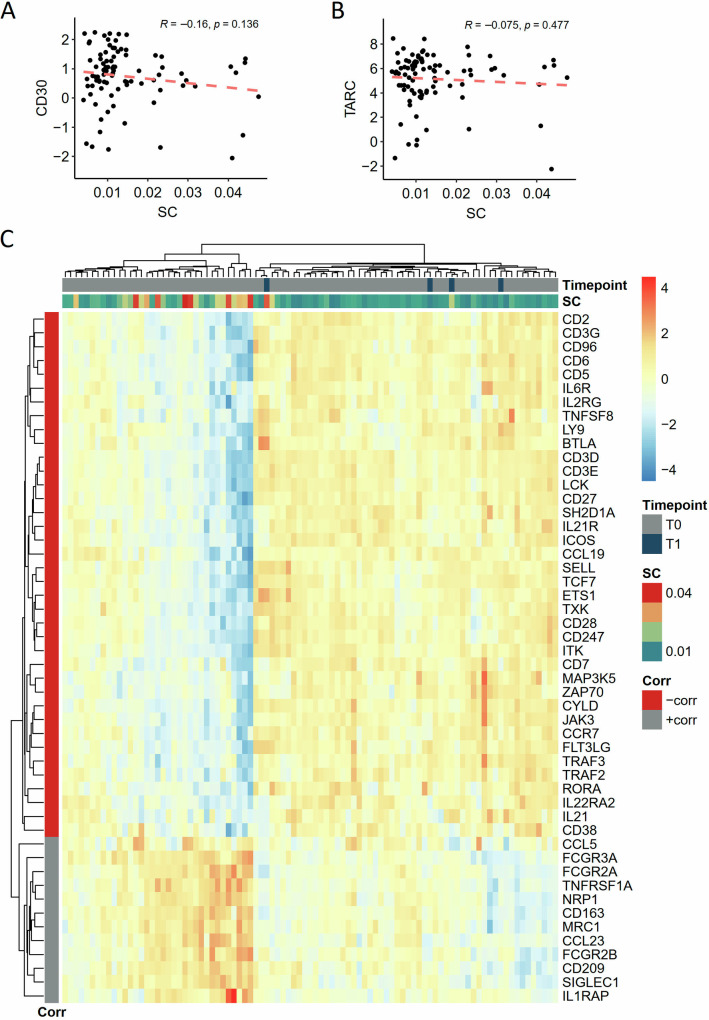
Correlation of T-cell clonality with gene expression in the tumor microenvironment of HL. Nanostring gene expression data previously published [8, 13]. **A** Correlation of CD30 expression with Simpson’s clonality (SC) in biopsies of treatment-naïve Hodgkin Lymphoma (HL, *n* = 87) and HL under ICB (subset of HL as paired biopsies under ICB, *n* = 4). **B** Correlation of TARC/CCL17 expression with SC in biopsies of treatment-naïve HL and HL under ICB as shown in (**A**). **C** Heatmap and two-dimension hierarchical clustering of gene expression levels in biopsies of treatment-naïve HL (T0) and HL under ICB (T1) as shown in (**A**), selected for the top 50 genes negatively (-corr) or positively correlated (+corr) with SC.

Despite the fact that the relative content of T-cell clones in the TME of HL is low, we searched for tissue features that are associated with clonality by gene expression profiling and immunohistochemistry using WSI analysis as previously described [17]. The group of genes negatively associated with clonality contained several pan-T-cell markers (e.g., CD3, CD2, CD5) suggesting that a high T-cell content leads to a relatively low clonality (relative low abundancy of clonal T-cells among all T-cells), a finding that was confirmed by quantitative immunohistochemistry (Supplementary Fig. 4). Inversely, genes positively associated with clonality reflected macrophage content (e.g., CD163, CD209, SIGLEC-1, Fig. 2C). However, quantitative analysis of CD68 or PDL1 immunohistochemistry reflecting marcophage content did not correlate with clonality (Supplementary Fig. 4). Similarly, expression of CD30 or TARC/CCL17 mRNA was not correlated with clonality (Fig. 2A, B, respectively) whereas quantitative immunohistochemistry did show an inverse correlation between CD30 expression and clonality (Supplementary Fig. 4). A relatively poor correlation between gene expression and WSI analysis has been observed by us previously mainly for large cell types such as macrophages and HRSC [1] and explains the discrepancy between gene expression and quantitative immunohistochemistry for these cell types. To understand the clinical significance of TCR clonality, we correlated with clinical features. Since in the NIVAHL cohort no relapses have occurred yet, we correlated with response after 4x Nivolumab, age, sex, clinical stage, presence of B Symptoms but did not detect any significant association (data not shown). We additionally analyzed the cases from the HD12 and HD15 trials (Supplementary Table 1A) using clonality as a continuous parameter and Cox regression analysis but did not detect an association with OS or PFS in this cohort (OS: *p* = 0.705, *n* = 114, total number of events = 22, hazard ratio = 0.6818, PFS: *p* = 0.0872, *n* = 114, total number of events = 29, hazard ratio = 3.3033).

**Fig. 3:**
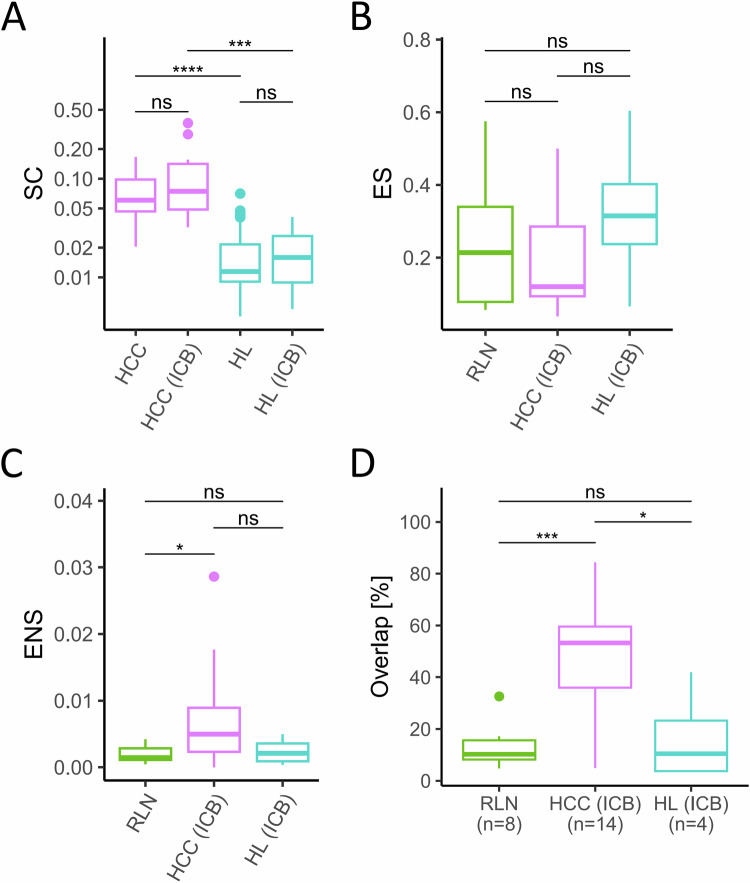
T-cell receptor (TCR) repertoire analysis in healthy tissue and in tumor microenvironments before and during/after immune checkpoint blockade. **A** Simpson’s Clonality (SC) in paired biopsies of patients with treatment naive hepatocellular carcinoma (HCC, (*n* = 14) and under immune checkpoint blockade (ICB, *n* = 14), and in biopsies of treatment-naïve Hodgkin Lymphoma (HL, (*n* = 90) and HL under ICB (subset of HL as paired biopsies under ICB, *n* = 4). **B** Clonal expansion of singletons (ES) between two biopsies of reactive lymph nodes (RLN, *n* = 8), and of HCC and HL pairs as shown in (**A**). **C** Clonal expansion of non-singletons (ENS) between two biopsies of RLN (*n* = 8), and of HCC and HL pairs as shown in (**A**). **D** Overlap of TCR sequences as percentage of TCR sequences with the same amino acid sequence in the treatment naive biopsy which were also detected in a follow-up biopsy of the same patient. Box and whiskers plots with median indicated as horizontal bar. ns not significant (*p* > 0.05), *p 0.05–0.01, **p < 0.01, ***p < 0.0001, ****p < 0.00001.

To gain further insight into the pathomechanism inhibiting clonal expansion of T-cells in the TME of HL and to distinguish anergy, inhibition, and exclusion, we analyzed bulk TCR sequences in sequential biopsies of HL and hepatocellular carcinoma (HCC) obtained during ICB, which is expected to unleash previously inhibited clonal expansion of T-cells. Sequential RLN biopsies served as a control of random expansion of TCR sequences over time. Neither in HCC nor HL an increase of clonality was detected when treatment naive biopsies were compared to specimens obtained during ICB (Fig. 3A). It is important to mention that re-biopsies of HL patients were obtained during the period of time when ICB exerts its clinical effects, i.e., in patients responding to anti-PD1-based first-line treatment [8]. Since clonality is already high in HCC prior to ICB (median SC = 0.0606), a maximum level of clonality may have already been reached in this solid cancer. In HL, however, clonality was significantly lower (median SC = 0.0115) than in HCC, but not further increased by ICB as reported previously [8] (Fig. 3A, *p* = <0.0001 prior and *p* = 0.001 under ICB). Next, we tested for expansion of singletons and non-singletons in sequential biopsies of HCC and HL during ICB to identify TCR repertoire dynamics. In HL and HCC, singletons did not expand at a significantly higher level compared to RLN when two sequential biopsies of the same patient were compared (Fig. 3B). In contrast, clonal expansion of non-singletons during ICB differed between HCC and HL. In comparison to control tissue, pre-expanded non-singletons were significantly more frequently expanded in HCC following ICB (Fig. 3C, *p* = 0.029), whereas no significant difference to RLN was observed in HL under ICB (Fig. 3C). Next, we analyzed the clone overlap in the treatment-naïve TME with the TME under ICB. As already observed for relapsed BC, the percentage of clones in the treatment-naïve TME that were also detected during ICB was significantly higher in HCC than in HL and RLN (Fig. 3D, *p* = 0.018 and *p* = 0.0006, respectively). Again, we observed no significant differences between HL and RLN. We hence conclude that, both conditions—re-exposure to the cancer at relapse or immune activation by ICB—are associated with re-occurrence of preexisting TCR clones and expansion of predominantly pre-amplified T-cell populations in the TME of solid cancers, but this phenomenon is not detectable in HL.

**Fig. 4:**
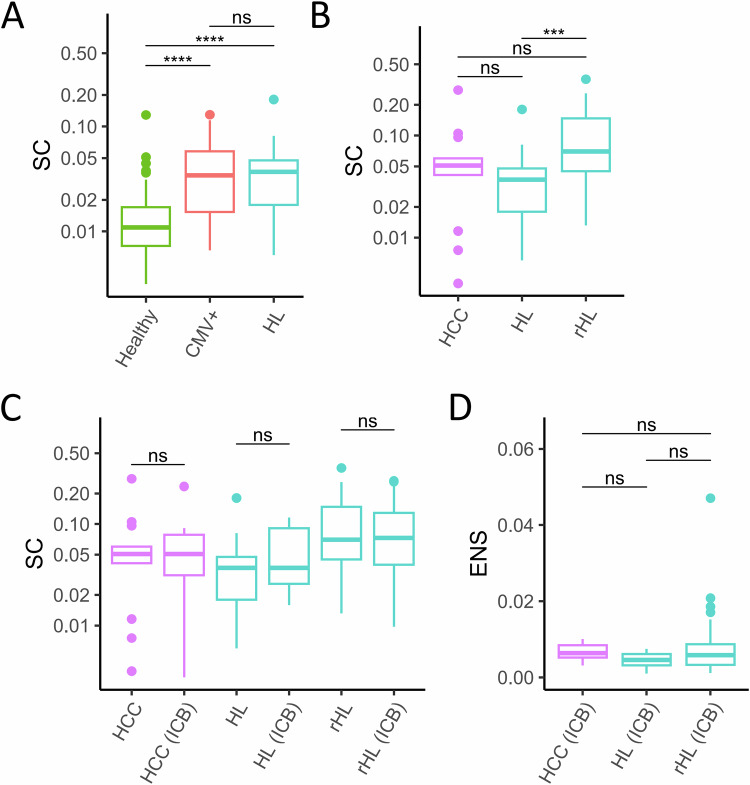
T-cell repertoire analysis in peripheral blood. **A** Simpson’s Clonality (SC) in PBMC of Healthy = healthy donors (*n* = 68), CMV+ = Cytomegalyvirus infected individuals (*n* = 51), HL = treatment-naive Hodgkin Lymphoma specimens (*n* = 21). **B** SC in peripheral blood mononuclear cells (PBMC) of HCC = hepatocellular carcinoma (*n* = 14), HL = treatment-naïve HL (*n* = 21), rHL = relapsed/refractory HL (*n* = 51). **C** SC in PBMC of HCC = paired samples of patients with hepatocellular carcinoma before immune checkpoint blockade (ICB, *n* = 14) and HCC (ICB) = under ICB (*n* = 14), HL = treatment-naïve HL specimens (*n* = 21) and HL (ICB) = subset of HL as paired samples during ICB (at final restaging, *n* = 8), rHL = paired samples of relapsed/refractory HL before ICB (*n* = 51) and rHL (ICB) = during ICB (after 4x Nivolumab, *n* = 45). **D** Clonal expansion of non-singletons (ENS) in PBMC of HCC, HL, and rHL pairs shown in (**C**). Box and whiskers plots with median indicated as horizontal bar. ns not significant (*p* > 0.05), *p 0.05–0.01, **p < 0.01, ***p < 0.0001, ****p < 0.00001.

To evaluate the contribution of exclusion of lymphoma-specific T-cells from the TME to the observed TCR polyclonality in the HL TME, we analyzed peripheral blood mononuclear cells (PBMC) of HL patients during ICB-based first-line treatment. First, we analyzed bulk PBMC containing all types of T-cells. As expected, healthy controls showed the lowest clonality (Fig. 4A, median SC = 0.0109). Bulk analysis of TCR repertoires in PBMC is obviously able to identify TCR expansion in immune reactions since CMV infected patients showed significantly higher clonality (median SC = 0.0342) than healthy controls (Fig. 4A, *p* = <0.0001). Interestingly, PBMC of treatment-naïve HL patients showed higher clonality (median SC = 0.0369) than healthy controls indicating clonal TCR expansions in the peripheral blood of HL patients before any therapy and in contrast to the findings in TME (*p* < 0.0001, Fig. 4A, PoS see Supplementary Fig. 5A). However, increased TCR clonality in the blood is not specific for HL patients since the PBMC of patients suffering from HCC displayed similarly high clonality (Fig. 4B, median SC = 0.0507) as reported previously [18]. Although the PBMC of treatment-naïve HL and those obtained at relapse are not derived from the same patients and therefore, it is not possible to determine non-singleton expansion, it still appears notable that clonality is higher at relapse (Fig. 4B, median SC = 0.0701, *p* = 0.0004, PoS see Supplementary Fig. 5B). In line with the findings in tissue biopsies, TCR clonality in PBMC did neither increase in treatment-naïve HL nor in relapsed HL during and after ICB (Fig. 3C, PoS see Supplementary Fig. 5C). Similar results were obtained when the time points during and after ICB were analyzed separately (Supplementary Table 5). Clonal expansion during and after ICB did not differ between treatment-naïve HL and relapsed HL (Fig. 4D and Supplementary Fig. 5D).

**Fig. 5:**
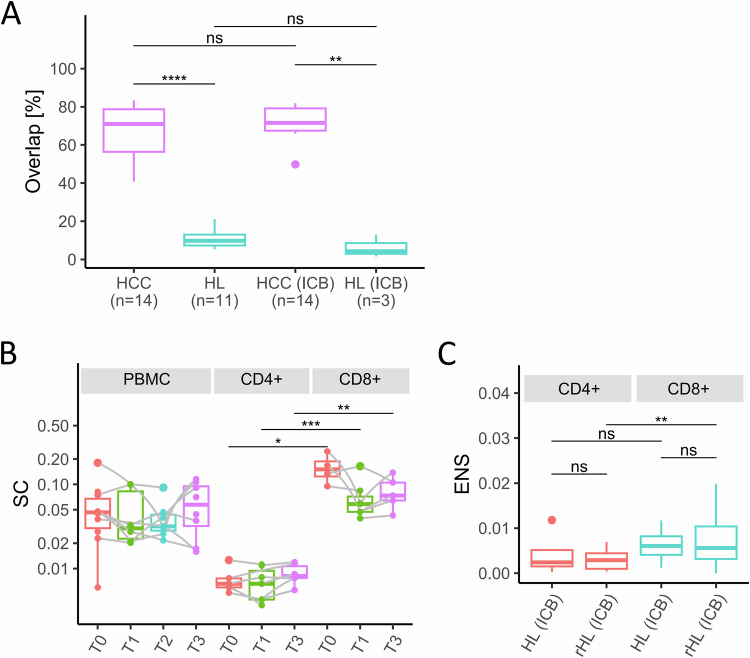
Overlap of T-cell receptor (TCR) sequences in the tumor microenvironment and the peripheral blood and T-cell receptor repertoire analysis of sorted cells in the peripheral blood. **A** Overlap of TCR amino acid sequences in paired samples of tumor microenvironment (TME) and peripheral blood mononuclear cells (PBMC) of treatment naive patients with hepatocellular carcinoma (HCC) and Hodgkin Lymphoma (HL) and of patients during/after immune checkpoint blockade (ICB). Overlap of TCR sequences as percentage of TCR sequences with the same amino acid sequence in the TME which were also detected in the peripheral blood of the same patient. **B** SC in PBMC and sorted CD4+ or CD8+ T-cell populations in patients enrolled in the NIVAHL trial ^13^T0 = treatment-naïve HL at diagnosis (PBMC *n* = 10, CD4+ *n* = 5, CD8+ *n* = 4), T1 = early on-treament, 1–2 weeks after start of treatment (PBMC *n* = 6, CD4+ *n* = 7, CD8+ *n* = 7), T2 = 1st restaging, after 2x Nivolumab-AVD or 4x Nivolumab (PBMC *n* = 8), T3 = at final restaging (PBMC *n* = 8, CD4+ *n* = 6, CD8+ *n* = 5). **C** Clonal expansion of non-singletons (ENS) in sorted CD4+ or CD8+ T-cell populations of HL and relapsed/refractory HL pairs. Box and whiskers plots with median indicated as horizontal bar. ns not significant (*p* > 0.05), *p 0.05–0.01, **p < 0.01, ***p < 0.0001, ****p < 0.00001.

The increase in clonality and decrease in PoS in the peripheral blood of HL patients at relapse may present increased expansion of T-cell populations upon re-exposure of the immune system to the lymphoma. The discrepancy in clonality between TME and blood suggests that T-cell expansions in HL patients may occur more effectively outside the TME at other sites of the body. However, in contrast to solid cancers, the clonally expanded T-cells do not seem to enter the TME efficiently, resulting in the observed discrepantly lower TCR clonality in the HL TME than the peripheral blood. To find more evidence for exclusion of clonally expanded peripheral blood T-cells from the TME, we analyzed the overlap of clones between tissue and peripheral blood. The percentage of TCR sequences in the TME that were also detected in the peripheral blood of the same patient was significantly higher in HCC compared to HL specimens (Fig. 5A, *p* < 0.0001 prior and *p* = 0.003 under ICB). Of note, the percentage of clone overlap did not increase during ICB for both entities (Fig. 5A).

To understand if T-cell subtypes may exhibit clonal expansion that is missed in bulk PBMC analyses, we used sorted CD4+ and CD8+ PBMC obtained before, during and after ICB as first-line therapy for HL. We observed increased clonality of CD8+ compared to CD4+ PBMC of HL patients at all time points (Fig. 5B, *p* = 0.016 before ICB, *p* = 0.0006 during ICB, *p* = 0.004 after ICB). However, clonality did not change in any T-cell type during ICB (Fig. 5B and Supplementary Table 5, PoS: Supplementary Fig. 6A). Thus, ICB does not induce obvious clonal expansion of CD4+ or CD8+ T-cells in peripheral blood during anti-PD1-based first-line treatment of HL patients. Re-analysis of an independent cohort of refractory/relapsed HL yielded similar results (Supplementary Fig. 7 and Supplementary Table 5). Analyzing the dynamic changes during ICB, we found that singletons did not expand differently in CD4+ and CD8+ cells (Supplementary Fig. 6B). In contrast, non-singletons expanded significantly higher in CD8+ compared to CD4+ cells (*p* = 0.0095, Fig. 5C). Thus, under ICB, clonal expansion in the blood of HL patients predominantly affects CD8+ cells and mostly pre-amplified T-cell populations (non-singletons).

## Discussion

The TME of HL contains abundant but also highly polyclonal T-cells. In this respect, HL differs strikingly from solid cancers. Certainly, polyclonality of T-cells observed in our bulk analysis does not rule out that smaller clones of T-cells are present in the HL TME as shown previously by single-cell analysis on a limited number of cases [15, 16, 19]. Our data show clonal T-cell in the TME in HL too. However, their relative frequency among all T-cells is rather low. Nevertheless, small clonal T-cell expansion certainly occur in the TME of HL too and these may in fact represent CD8+ T-cells [15, 16, 19].

At least the largest fraction if not even the majority of T-cells in the TME of HL are unlikely part of an anti-lymphoma immune response as they are highly diverse. Since HRSC secrete high levels of cytokines, they likely actively attract T-cells to the TME independent of their specificity. Once present in the TME, these T-cells appear to remain polyclonal in contrast to T-cells in solid cancers. The persistent block of clonal expansion in the TME of HL may be explained by anergy mediated through HRSC due to lack of HLA-molecules on their cell surface [20] and local inhibition of T-cell expansion via activation of immune checkpoint molecules such as PD1 or LAG3 axis [9, 21]. The relative contribution of these two potential mechanisms for the prevention of clonal T-cell expansion is difficult to study without single-cell data. However, our data derived from clinically well-annotated and contemporarily treated longitudinal cohorts, including anti-PD1-based first-line treatment, may provide some insight into this phenomenon:

If HRSC are anergic to T-cells, ICB should not lead to a clonal expansion since T-cells are simply not detecting an antigen presented by an HLA molecule. In contrast, inhibition of T-cells by HRSC is expected to be overcome by anti-PD1 ICB and the latter should lead to clonal expansion of T-cells. Since we do not detect clonal expansion of T-cells during ICB, anergy of HRSC may in fact be a relevant mechanism that prevents clonal expansion of T-cells in the TME of HL. However, local inhibition of T-cells by HRSC cannot completely be excluded as a contributing mechanism since the number of early re-biopsies of HL during ICB is limited. Additionally, the time frame of 1–15 days between initiation of ICB and re-biopsy in the available cohorts [8] is rather short to allow for the mounting of a clonally expanded T-cell response. Our analysis may thus miss a later occurring clonal T-cell expansion during ICB. Nevertheless, the striking early clinical effect in the enlarged lymph nodes seems to occur without detectable clonal expansion of pre-amplified T-cell clones in the TME of HL. The fact that expansion of pre-amplified T-cell populations (non-singletons) is lacking in HL, whereas it is detectable in solid cancer patients at relapse suggests that even tumor-specific T-cells are prevented from local expansion within the TME.

Our data suggest to include another mechanism in addition to anergy and inhibition to suppress an effective anti-lymphoma T-cell immune response. In solid cancers, most resident T-cells are irreversibly exhausted and do not start to proliferate with ICB. Instead T-cells activated at tumor-extrinsic sites expand and populate the TME of non-hematological cancers [22, 23]. In fact, even in solid tumors, a relevant number of T-cells within the TME recognizes nonspecific (e.g., viral) antigens [24]. We herein detected a moderate increase of clonality in the TME of HL at relapse. This increase of clonality reflects a decreased diversity of T-cells at relapse that arises without expansion of T-cells pre-amplified in the primary biopsy (non-singletons). Since clonality is increased in the peripheral blood T-cells of HL patients at first diagnosis and even more so at relapse, we assume that T-cells are clonally expanded, however, mostly outside of the TME. These clonally expanded T-cells enter, at least partially, the TME of relapsed HL, leading to slightly increased clonality but may then be locally prevented from further expansion or an effective anti-tumor immune response. The infiltration of the TME by T-cells of lower diversity/higher clonality is detectable at relapse of HL but clonality levels still remain below those found in solid cancers. Thus, even T-cells which expanded at tumor-external sites enter the HL TME inefficiently. These findings suggest other mechanisms in addition to anergy and inhibition to contribute to polyclonality of T-cells in HL. We suggest that cellular exclusion of T-cells from the TME may be a major contributor to polyclonality as exemplified by (i) a marked discrepancy in clonality between blood and TME and (ii) a low overlap of TCR sequences between blood and TME in HL patients. In this respect HL seems to differ from solid cancers, where clonally expanded T-cells appear to enter the TME effectively, clonality shows no discrepancy between blood and TME and higher overlap of clones between peripheral blood and TME is observed. The mechanism how T-cells clonally expanded in the blood are excluded from the TME of HL remains uncertain. To the best of our knowledge, there is no cytokine condition that actively prevents the migration of specific T-cells to the tumor. Thus, one might speculate that cytokines produced by HRSC actively attract abundant but random T-cells from the bloodstream of which most are naturally not tumor specific. This may lead to a dilution of tumor-specific clones within the TME and explain the differences to solid tumors (exclusion by dilution).

In this study, we used TCR repertoire analysis to understand T-cell diversity, clonal expansion, and dynamics in HL TME and blood specimens using more than 700 specimens and almost 78 million TCR sequences. To understand the dynamics of the T-cells solely based on TCR sequencing, we studied (i) primary and relapse specimens without ICB exposure as a model for re-exposure of the immune system to the tumor (ii) biopsies obtained during ICB to understand the contribution of immune checkpoints and their inhibition and (iii) paired PBMC and TME specimens to describe tumor external, systemic T-cell expansion. While this constitutes the most comprehensive TCR analysis of HL so far, several questions remain unresolved due to the mostly descriptive nature of our data. Since non-affected sites are rarely biopsied in these patients, it is not possible to track TCR clones between the TME, peripheral blood, and other sites. Moreover, the specificity of clonally expanded T-cells against HRSC awaits functional proof. To date, it remains uncertain to what extent the clonality of T-cells reflects a specific anti-lymphoma immune response [25]. TCR sequences linked to epitopes expected in HRSC such as EBV proteins and cancer testis antigens can only be inferred from the available databases, which appear to contain too few annotations for definite conclusions at the current time.

Analyzing TME features associated with clonality we found the T-cell content to be inversely correlated with clonality. This finding suggests, that abundant T-cells in the TME do not represent an anti-lymphoma immune response which should be reflected by clonal expansion. However, larger cohorts that combine TCR repertoire analysis with TME feature assessment are required to define the TME features that associate with clonal expansion of T-cells in the TME.

Finally, the clinical relevance of clonality warrants further studies. Since in the NIVAHL trial, no events have been observed yet, clinical correlations are not feasible. Our cohort from HD12/15 did not show a significant association of clonality with overall or progression-free survival. However, this cohort is probably not large enough for definite conclusions. Anyhow, it is questionable if clonality can be expected to be a biomarker of survival under conventional polychemotherapy.

To fully understand the contribution of anergy, inhibition, and exclusion to the TCR polyclonality observed in the TME, larger cohorts of clinically well-annotated, contemporarily treated paired samples of PBMCs, HL biopsies, and healthy (ideally lymphoid) tissue are needed. Most importantly, additional single-cell studies from these different compartments are required to identify the exact clonal structure of the T-cells in HL patients. Additionally, further analyses of early on-ICB-treatment will be crucial to study the effect of ICB on T-cell repertoire and function in HL. Due to the limited sample size findings in HL and solid cancer specimen required confirmation. Furthermore, biopsies of HL relapsed after ICB-based first-line treatment are exceedingly rare [26] hampering comparative analyses of the specific effect of ICB on T-cell expansion compared to conventional chemotherapy at relapse. Finally, it remains uncertain how exclusion of clonally expanded T-cells from the TME contributes to the apparent immune evasion of HL. Herein, we lay important groundwork for future study to address these important questions and dissect local and systemic T-cell trajectories in HL. Our data suggest that exclusion exists but the extent and underlying mechanisms in certain subtypes of HL need to be studied. Such insights are crucial to further unlock the tremendous therapeutic potential of ICB in HL to further reduce exposure to conventional chemo- and radiotherapy and address unmet needs in frail or high-risk HL patients.

## Supplementary information

Supplemental Material

## Data Availability

Materials described in the manuscript, including all relevant raw data, will be freely available to any researcher wishing to use them for non-commercial purposes, without breaching participant confidentiality. The TCR data deposition for download is indicated in Supplementary Tables 1A and 2.
