# Supplementary material for: T-cell diversity and exclusion of blood-derived T-cells in the tumor microenvironment of classical Hodgkin Lymphoma
Source: Leukemia. 2024 Dec 17;39(3):684–93. doi: 10.1038/s41375-024-02490-6 (PMC11879864; doi:10.1038/s41375-024-02490-6)
Supplement: Supplementary file 1 — Supplemental Material [file 41375_2024_2490_MOESM1_ESM.docx]

**Supplementary Methods**

Antigen distributions were calculated for each sample as follows: First, the easy-search function of MMSeqs2^1^ was used to align all clones against the TCR-pMHC database VDJdb.^2^ Only alignments with an E-value ≤ 0.05 were kept. Clones with several database matches were further filtered for best alignment: first by lowest E-value, then by highest bit-score and finally, by highest sequence identity. If the remaining database matches were annotated with the same target antigen, the clone was also annotated with respective target antigen. If target antigens were not identical, the clone received no annotation. Proportions of annotated antigens were calculated for each sample by dividing the count-sum of all clones with the same annotation by the count-sum of all clones with any annotation. Dynamic changes in antigen distribution, e.g. during ICB, were assessed by calculating the fold change in antigen frequencies following treatment.

**Supplementary Tables**

**Supplementary Table 1A: Cohorts with TCR repertoires of tissue biopsies**

| **Cohort** | **Timepoint** | **Description** | **Patients** | **Origin** |
| --- | --- | --- | --- | --- |
| RLN | T0 | reactive lymph nodes | n=8 | Diagnostic cases from Department of Pathology, University Hospital of Kiel (UKSH), Germany |
| RLN (2nd) | T1 | RLN subsequent biopsy (details see below) | n=8 (paired) |  |
| BC | T0 | treatment-naive breast cancer | n=6 | Diagnostic cases from Department of Pathology, UKSH, Germany |
| BC (relapse) | T1 | BC relapse after surgery (details see below) | n=6 (paired) |  |
| HCC | T0 | hepatocellular carcinoma previously treated with multi-tyrosine kinase inhibitor (mTKI) | n=14 | [immuneACCESS (DOI: 10.21417/RP2024NM)](https://doi.org/10.21417/RP2024NM) ^3^ |
| HCC (ICB) | T1 | HCC following second-line ICB (Pembrolizumab & neo-antigen vaccine, 9 weeks after start of therapy) | n=14 (paired) |  |
| HL | T0 | treatment-naive Hodgkin lymphoma | n=108 | NIVAHL^4^ (DOI: 10.1001/jamaoncol.2020.0750, n=90), diagnostic cases from Department of Pathology, UKSH, Germany (n=18) |
| HL (ICB) | T1 | HL during first-line ICB (1-75 days after start of Nivolumab or Nivolumab-AVD) | n=4 (paired) | NIVAHL^4^ (DOI: 10.1001/jamaoncol.2020.0750) |
| HL (relapse) | T1 | HL relapse after conventional chemotherapy (details see below) | n=18 (paired) | Diagnostic cases from German Lymphoma Reference Pathology, Germany ^5^ |
| HD12/15 | T0 | treatment naive advanced stage HL | n=81 | GHSG HD12 and HD15 studies ^6^ |

Abbreviations: RLN: reactive lymph nodes, BC: breast cancer, HCC: hepatocellular carcinoma, HL: classical Hodgkin lymphoma, ICB: immune-checkpoint blockade. Paired: follow-up samples of previous biopsies from the same patients.
HD12/15 TCR repertoires were sequenced by Amplicon TRBVJ next-generation sequencing of lymphoma tissue as described previously.^7^ Lymphoma specimens were analyzed with the ARResT/Interrogate immunoprofiler (arrest.tools/interrogate)^8^ and further filtered for productive rearrangements.

**Supplementary Table 1B: Details for cohorts of paired samples**

| **Cohort HL Paired** | **Sex** | **Age (years)** | **Localisation Primary** | **Localisation Relapse** | **Time between Primary and Relapse** |
| --- | --- | --- | --- | --- | --- |
| From:  Schnitter et al. | m | 41 | cervical LN left | axcillar LN right | 53 months 25 days |
|  | m | 33 | retroperitoneal | LN | 53 months 30 days |
|  | m | 22 | LN | supraclavicular LN right | 11 months 22 days |
|  | f | 74 | LN | cervical LN left | 44 months 6 days |
|  | m | 16 | cervical LN right | Mediastinum (lymphatic) | 12 months |
|  | m | 28 | cervical LN left & right | LN | 16 months 15 days |
|  | f | 64 | cervical LN right | LN | 16 months 7 days |
|  | m | 28 | axcillar LN right | paraaortal LN | 98 months 10 days |
|  | f | 16 | cervical LN left | axcillar LN left | 21 months 3 Tagw |
|  | m | 37 | LN | lymphatic tissue | 66 months 29 days |
|  | m | 67 | LN left upper tight | upper tight | 6 months 18 days |
|  | m | 24 | cervical LN | cervical LN left | 34 months 21 days |
|  | m | 27 | LN | supraclavicular LN right | 30 months 0 days |
| Additional  diagnostic  cases | m | 62 | groin LN | axcilla LN | 29 months 5 days |
|  | m | 56 | supraclavicular LN right | paraaortal LN | 100 months 1 days |
|  | f | 29 | cervical LN left | cervical LN right | 9 months 20 days |
|  | m | 71 | cervical LN | cervical LN left | 21 months 9 days |
|  | f | 30 | cervical LN right | LN | 7 months 27 days |
| **Cohort RLN - Paired** | **Sex** | **Age (years)  at first diagnose** | **Localisation Primary** | **Localisation Relapse** | **Time between Primary and Relapse** |
|  | f | 29 | cervical LN left | cervical LN | 28 months 10 days |
|  | f | 19 | submandibular LN right | LN jaw angle right | 21 months 14 days |
|  | m | 38 | inguinal LN left | inguinal LN left | 16 months 1 day |
|  | f | 57 | cervical LN neck right | submandibular LN | 4 months 26 days |
|  | m | 13 | LN jaw angle right | axcillar LN left | 32 months 26 days |
|  | m | 42 | intra-axcillar LN left | LN jaw angle right | 8 months 15 days |
|  | f | 63 | inguinal LN left | groin LN left | 7 months 19 days |
|  | m | 20 | axcillar LN left | groin LN left | 28 months 20 days |
| **Cohort BC - Paired** | **Sex** | **Age (years)** | **Time between Primary and Relapse** |  |  |
|  | f | 62 | 112 months 4 days |  |  |
|  | f | 54 | 44 months 3 days |  |  |
|  | f | 39 | 25 months 8 days |  |  |
|  | f | 87 | 16 months 30 days |  |  |
|  | f | 34 | 73 months 12 days |  |  |
|  | f | 43 | 30 months 25 days |  |  |

Abbreviations: RLN: reactive lymph nodes, BC: breast cancer, HL: classical Hodgkin lymphoma, Paired: follow-up samples of previous biopsies from the same patients, m: male, f: female, LN: Lymph node.

**Supplementary Table 2: Cohorts with TCR repertoires of blood samples**

| **Cohort** |  | **Timepoint** | **Description** | **Cell type** | **Patients** | **Origin** |
| --- | --- | --- | --- | --- | --- | --- |
| Healthy |  | T0 | healthy individuals | PBMC | n=68 | immuneACCESS (DOI: [10.21417/B7001Z](https://doi.org/10.21417/B7001Z), Cohort 2) ^9^ |
| CMV+ |  | T0 | recent CMV-infection but otherwise healthy | PBMC | n=51 |  |
| HCC |  | T0 | hepatocellular carcinoma (treated with multi-tyrosine kinase inhibitor) | PBMC | n=14 | [immuneACCESS (DOI: 10.21417/RP2024NM)](https://doi.org/10.21417/RP2024NM) ^3^ |
| HCC (ICB) |  | T1 | HCC following second-line ICB (Pembrolizumab & neo-antigen vaccine, 6-21 weeks after start of therapy) | PBMC | n=14 (paired) |  |
| HL |  | T0 | treatment-naive Hodgkin lymphoma | PBMC | n=21 | NIVAHL^4^ (DOI: 10.1001/jamaoncol.2020.0750, n=10), immuneACCESS ([DOI: 10.21417/FZC2020NM](https://doi.org/10.21417/FZC2020NM), n=11) |
|  |  |  |  | CD4+ | n=16 | NIVAHL^4^ (DOI: 10.1001/jamaoncol.2020.0750, n=5), immuneACCESS ([DOI: 10.21417/FZC2020NM](https://doi.org/10.21417/FZC2020NM), n=11) |
|  |  |  |  | CD8+ | n=15 | NIVAHL^4^ (DOI: 10.1001/jamaoncol.2020.0750, n=4), immuneACCESS ([DOI: 10.21417/FZC2020NM](https://doi.org/10.21417/FZC2020NM), n=11) |
| HL (ICB) |  | T1 | HL during first-line ICB (early on-treament, 1-2 weeks after SOT) | PBMC | n=6 (paired) | NIVAHL^4^ (DOI: 10.1001/jamaoncol.2020.0750) |
|  |  |  |  | CD4+ | n=7 (paired) |  |
|  |  |  |  | CD8+ | n=7 (paired) |  |
|  |  | T2 | HL during first-line ICB (1st restaging after 2x Nivolumab-AVD or 4x Nivolumab) | PBMC | n=8 (paired) |  |
|  |  |  |  | CD4+ | - |  |
|  |  |  |  | CD8+ | - |  |
|  |  | T3 | HL during first-line ICB (final restaging) | PBMC | n=8 (paired) |  |
|  |  |  |  | CD4+ | n=6 (paired) |  |
|  |  |  |  | CD8+ | n=5 (paired) |  |
| rHL |  | T0 | relapsed/refractory Hodgkin lymphoma | PBMC | n=51 | immuneACCESS ([DOI: 10.21417/FZC2020NM](https://doi.org/10.21417/FZC2020NM))^10^ |
|  |  |  |  | CD4+ | n=20 |  |
|  |  |  |  | CD8+ | n=20 |  |
| rHL (ICB) |  | T1 | rHL during second-line ICB (after 2x Nivolumab) | PBMC | n=46 (paired) |  |
|  |  |  |  | CD4+ | - |  |
|  |  |  |  | CD8+ | - |  |
|  |  | T2 | rHL during second-line ICB (after 4x Nivolumab) | PBMC | n=45 (paired) |  |
|  |  |  |  | CD4+ | n=20 (paired) |  |
|  |  |  |  | CD8+ | n=20 (paired) |  |

Abbreviations: CMV: cytomegalyvirus, HCC: hepatocellular carcinoma, (rr)HL: (relapsed or refractory) classical Hodgkin lymphoma, ICB: immune-checkpoint blockade. Paired: follow-up samples of previous blood samples from the same patients.

| **Sample** | **Cohort** | **Patients** | **Min** | **Max** | **Std** | **Median** | **Sum** |
| --- | --- | --- | --- | --- | --- | --- | --- |
| Tissue | RLN | n=8 | 10546 | 102600 | 35019,7 | 44931 | 399313 |
|  | RLN (2nd) | n=8 | 2812 | 236879 | 79333,76 | 51645,5 | 572344 |
|  | BC | n=6 | 3103 | 71162 | 24260,73 | 18734 | 142302 |
|  | BC (relapse) | n=6 | 8598 | 55130 | 15283,74 | 26253 | 164125 |
|  | HCC | n=14 | 122 | 24655 | 6719,82 | 5971 | 91142 |
|  | HCC (ICB) | n=14 | 140 | 38148 | 12383,33 | 6382 | 172092 |
|  | HL | n=108 | 1164 | 270527 | 60614,65 | 51865 | 7467469 |
|  | HL (ICB) | n=4 | 32785 | 314422 | 123362,6 | 100518 | 548243 |
|  | HL (relapse) | n=18 | 1588 | 156976 | 43510,42 | 17886 | 574240 |
|  | HD12/15 | n=81 | 32 | 809600 | 128304,28 | 22517 | 5457296 |
| Blood (PBMC) | Healthy | n=68 | 72648 | 794743 | 179471,1 | 236705 | 19048955 |
|  | CMV+ | n=51 | 44656 | 815687 | 195765,8 | 286032 | 16845164 |
|  | HCC | n=14 | 129873 | 325987 | 50614,11 | 250603 | 3354557 |
|  | HCC (ICB) | n=14 | 102244 | 330129 | 67927,68 | 237027 | 3236538 |
|  | HL | n=21 | 11167 | 109867 | 24257,53 | 36467 | 922565 |
|  | HL (ICB, T1) | n=6 | 25083 | 121584 | 38589,37 | 65065 | 440369 |
|  | HL (ICB, T2) | n=7 | 26812 | 83817 | 17390,29 | 59922 | 416994 |
|  | HL (ICB, T3) | n=8 | 19044 | 147856 | 39361,34 | 51658 | 474627 |
|  | rHL | n=51 | 1860 | 260958 | 54968,23 | 54836 | 3816744 |
|  | rHL (ICB, T1) | n=46 | 16458 | 459687 | 80958,62 | 79213,5 | 5002402 |
|  | rrHL (ICB, T2) | n=45 | 21698 | 263703 | 64297,93 | 82184 | 4439078 |
| Blood (CD4+) | HL | n=16 | 5263 | 107684 | 27099,85 | 37649,5 | 701912 |
|  | HL (ICB, T1) | n=7 | 68569 | 103729 | 14817,88 | 78308 | 586114 |
|  | HL (ICB, T3) | n=6 | 24509 | 94894 | 31932,12 | 49674 | 341331 |
|  | rHL | n=20 | 1389 | 48920 | 15142,8 | 14278 | 381528 |
|  | rHL (ICB, T2) | n=20 | 4977 | 49032 | 14984,04 | 34928 | 644825 |
| Blood (CD8+) | HL | n=15 | 3858 | 83134 | 25368,29 | 14488 | 380436 |
|  | HL (ICB, T1) | n=7 | 28474 | 81218 | 22034,13 | 40595 | 354039 |
|  | HL (ICB, T3) | n=5 | 3798 | 84620 | 31728,11 | 20962 | 173229 |
|  | rHL | n=20 | 1295 | 48263 | 14745,44 | 14255,5 | 347150 |
|  | rHL (ICB, T2) | n=20 | 6410 | 36520 | 9726,58 | 26413,5 | 492822 |
| **All cohorts combined:** | | **n=734** | **32** | **815687** | **135758,2** | **56787,5** | **77989945** |

**Supplementary Table 3: Overview of the size of TCR repertoires (number of sequenced CDR3 regions)** **across cohorts.**

Abbreviations: Min= size of smallest TCR repertoire of a cohort, Max= size of biggest TCR repertoire of a cohort, Std= standard deviation of the sizes of all TCR repertoires in a cohort, Median= median size of a TCR repertoire of a cohort, Sum= total number of CDR3 sequences in all samples of a cohort.

| **Timepoint** | **group1** | **group2** | **n1** | **n2** | **p** | **FDR** | **p.signif** |
| --- | --- | --- | --- | --- | --- | --- | --- |
| T0 | RLN | BC | 8 | 6 | 0,000666 | 0,000999 | *** |
|  |  | HCC | 8 | 14 | 6,25E-06 | 1,88E-05 | **** |
|  |  | HL | 8 | 108 | 0,014 | 0,017 | * |
|  | BC | HCC | 6 | 14 | 0,076 | 0,076 | ns |
|  |  | HL | 6 | 108 | 0,000321 | 0,000642 | *** |
|  | HCC | HL | 14 | 108 | 3,19E-08 | 1,91E-07 | **** |
| T1 | RLN (2nd) | BC (relapse) | 8 | 6 | 0,001 | 0,003 | *** |
|  |  | HCC (ICB) | 8 | 14 | 6,25E-06 | 6,25E-05 | **** |
|  |  | HL (ICB) | 8 | 4 | 0,57 | 0,57 | ns |
|  |  | HL (relapse) | 8 | 18 | 0,000241 | 0,000803 | *** |
|  | BC (relapse) | HCC (ICB) | 6 | 14 | 0,02 | 0,033 | * |
|  |  | HL (ICB) | 6 | 4 | 0,114 | 0,143 | ns |
|  |  | HL (relapse) | 6 | 18 | 0,047 | 0,067 | * |
|  | HCC (ICB) | HL (ICB) | 14 | 4 | 0,001 | 0,003 | *** |
|  |  | HL (relapse) | 14 | 18 | 2,97E-05 | 0,000148 | **** |
|  | HL (ICB) | HL (relapse) | 4 | 18 | 0,227 | 0,252 | ns |

**Supplementary Table 4: Statistical comparison of Simpson’s Clonality (SC) in tissue biopsies**

Assessed by Wilcoxon rank-sum test. N1= sample size of cohort defined in group1, n2= samples size cohort defined in group2, FDR = all p-values (n=16) adjusted for multiple testing using Benjamini-Hochberg procedure, resulting in false-discovery rate, p.signif= ns: not significant (p>0.05), * p 0.05-0.01, ** p<0.01, *** p <0.0001, **** p<0.00001.

**Supplementary Table 5: Statistical comparison of Simpson’s Clonality (SC) in blood samples**

| **timepoint** | **group1** | **group2** | **n1** | **n2** | **p** | **FDR** | **p.signif** |
| --- | --- | --- | --- | --- | --- | --- | --- |
| T0 | CMV+ PBMC | HCC PBMC | 51 | 14 | 0,205 | 0,217 | ns |
|  |  | healthy control PBMC | 51 | 68 | 2,17E-08 | 1,12E-07 | **** |
|  |  | HL CD4+ | 51 | 16 | 3,76E-07 | 1,35E-06 | **** |
|  |  | HL CD8+ | 51 | 15 | 0,000115 | 0,00023 | *** |
|  |  | HL PBMC | 51 | 21 | 0,872 | 0,872 | ns |
|  |  | rHL CD4+ | 51 | 20 | 0,059 | 0,071 | ns |
|  |  | rHL CD8+ | 51 | 20 | 1,9E-07 | 7,6E-07 | **** |
|  |  | rHL PBMC | 51 | 51 | 2,89E-06 | 7,43E-06 | **** |
|  | HCC PBMC | healthy control PBMC | 14 | 68 | 0,000137 | 0,00026 | *** |
|  |  | HL CD4+ | 14 | 16 | 0,000185 | 0,000333 | *** |
|  |  | HL CD8+ | 14 | 15 | 0,057 | 0,071 | ns |
|  |  | HL PBMC | 14 | 21 | 0,154 | 0,168 | ns |
|  |  | rHL CD4+ | 14 | 20 | 0,02 | 0,029 | * |
|  |  | rHL CD8+ | 14 | 20 | 0,002 | 0,003 | ** |
|  |  | rHL PBMC | 14 | 51 | 0,073 | 0,084 | ns |
|  | healthy control PBMC | HL CD4+ | 68 | 16 | 0,033 | 0,044 | * |
|  |  | HL CD8+ | 68 | 15 | 7,42E-09 | 4,45E-08 | **** |
|  |  | HL PBMC | 68 | 21 | 3,43E-06 | 8,23E-06 | **** |
|  |  | rHL CD4+ | 68 | 20 | 0,005 | 0,007 | ** |
|  |  | rHL CD8+ | 68 | 20 | 5,15E-11 | 9,27E-10 | **** |
|  |  | rHL PBMC | 68 | 51 | 9,16E-17 | 3,3E-15 | **** |
|  | HL CD4+ | HL CD8+ | 16 | 15 | 6,65E-09 | 4,45E-08 | **** |
|  |  | HL PBMC | 16 | 21 | 6,93E-07 | 2,27E-06 | **** |
|  |  | rHL CD4+ | 16 | 20 | 5,94E-05 | 0,000126 | **** |
|  |  | rHL CD8+ | 16 | 20 | 2,74E-10 | 3,29E-09 | **** |
|  |  | rHL PBMC | 16 | 51 | 3,87E-09 | 3,48E-08 | **** |
|  | HL CD8+ | HL PBMC | 15 | 21 | 0,000369 | 0,000604 | *** |
|  |  | rHL CD4+ | 15 | 20 | 6,94E-06 | 1,56E-05 | **** |
|  |  | rHL CD8+ | 15 | 20 | 0,086 | 0,097 | ns |
|  |  | rHL PBMC | 15 | 51 | 0,713 | 0,733 | ns |
|  | HL PBMC | rHL CD4+ | 21 | 20 | 0,021 | 0,029 | * |
|  |  | rHL CD8+ | 21 | 20 | 1,49E-06 | 4,47E-06 | **** |
|  |  | rHL PBMC | 21 | 51 | 0,00036 | 0,000604 | *** |
|  | rHL CD4+ | rHL CD8+ | 20 | 20 | 6,53E-08 | 2,94E-07 | **** |
|  |  | rHL PBMC | 20 | 51 | 1,69E-06 | 4,68E-06 | **** |
|  | rHL CD8+ | rHL PBMC | 20 | 51 | 0,035 | 0,046 | * |
| T1 | HCC (ICB) PBMC | HL (ICB) CD4+ | 14 | 7 | 0,002 | 0,004 | ** |
|  |  | HL (ICB) CD8+ | 14 | 7 | 0,488 | 0,61 | ns |
|  |  | HL (ICB) PBMC | 14 | 6 | 0,718 | 0,729 | ns |
|  |  | rHL (ICB) PBMC | 14 | 46 | 0,146 | 0,243 | ns |
|  | HL (ICB) CD4+ | HL (ICB) CD8+ | 7 | 7 | 0,000583 | 0,003 | *** |
|  |  | HL (ICB) PBMC | 7 | 6 | 0,001 | 0,004 | *** |
|  |  | rHL (ICB) PBMC | 7 | 46 | 1,3E-08 | 1,3E-07 | **** |
|  | HL (ICB) CD8+ | HL (ICB) PBMC | 7 | 6 | 0,234 | 0,334 | ns |
|  |  | rHL (ICB) PBMC | 7 | 46 | 0,729 | 0,729 | ns |
|  | HL (ICB) PBMC | rHL (ICB) PBMC | 6 | 46 | 0,114 | 0,228 | ns |
| T2 | HL (ICB) PBMC | rHL (ICB) CD4+ | 7 | 20 | 0,000739 | 0,001 | *** |
|  |  | rHL (ICB) CD8+ | 7 | 20 | 0,002 | 0,002 | ** |
|  |  | rHL (ICB) PBMC | 7 | 45 | 0,052 | 0,052 | ns |
|  | rHL (ICB) CD4+ | rHL (ICB) CD8+ | 20 | 20 | 3,94E-08 | 1,18E-07 | **** |
|  |  | rHL (ICB) PBMC | 20 | 45 | 1,02E-08 | 6,12E-08 | **** |
|  | rHL (ICB) CD8+ | rHL (ICB) PBMC | 20 | 45 | 0,03 | 0,037 | * |
| T3 | HL (ICB) CD4+ | HL (ICB) CD8+ | 6 | 5 | 0,004 | 0,006 | ** |
|  |  | HL (ICB) PBMC | 6 | 8 | 0,000666 | 0,002 | *** |
|  | HL (ICB) CD8+ | HL (ICB) PBMC | 5 | 8 | 0,435 | 0,435 | ns |

Assessed by Wilcoxon rank-sum test. N1= sample size of cohort defined in group1, n2= samples size cohort defined in group2, FDR = all p-values (n=55) adjusted for multiple testing using Benjamini-Hochberg procedure, resulting in false-discovery rate, p.signif= ns: not significant (p>0.05), * p 0.05-0.01, ** p<0.01, *** p <0.0001, **** p<0.00001.

**Supplementary Figures**

**Supplementary Figure 1:** T-cell receptor repertoire analysis in healthy tissue and in tumor microenvironments of different cancers at primary diagnosis and relapse.


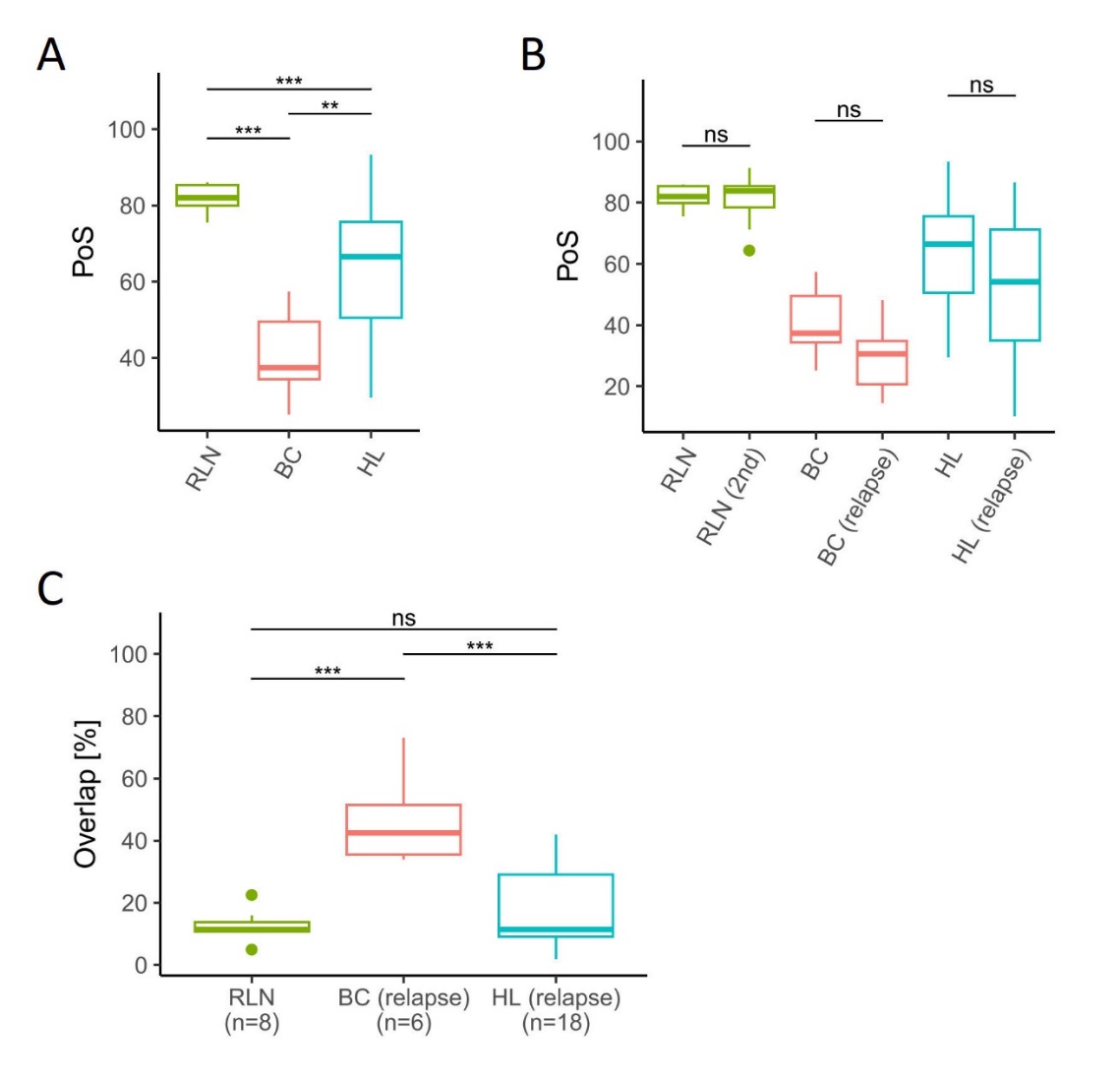


1. Percentage of Singletons (PoS) in reactive lymph nodes (RLN, n=8), treatment-naïve breast cancer (BC, n=6), treatment-naïve Hodgkin lymphoma (HL, n=108).
2. Percentage of Singletons (PoS) in paired biopsies of patients with treatment naive hepatocellular carcinoma (HCC, (n=14) and under immune checkpoint blockade (ICB, n=14), and in biopsies of treatment-naïve HL specimens (n=90) and HL under ICB (subset of HL as paired biopsies under ICB, n=4). SC in paired biopsies of patients with RLN as shown in A and a subsequent biopsy (RLN 2^nd^, n=8), of treatment-naïve BC as shown in A and BC at relapse after surgery (n=6), of treatment-naïve HL specimens as shown in A and HL at relapse (subset of HL as paired biopsies at relapse after chemotherapy, n=18).
3. Overlap of TCR sequences as percentage of TCR sequences with the same amino acid sequence in the follow-up biopsy which were also detected in the treatment naive biopsy of the same patient.

Box and whiskers plots with median indicated as horizontal bar. ns= not significant (p>0.05), * p 0.05-0.01, ** p<0.01, *** p <0.0001, **** p<0.00001

**Supplementary Figure 2**


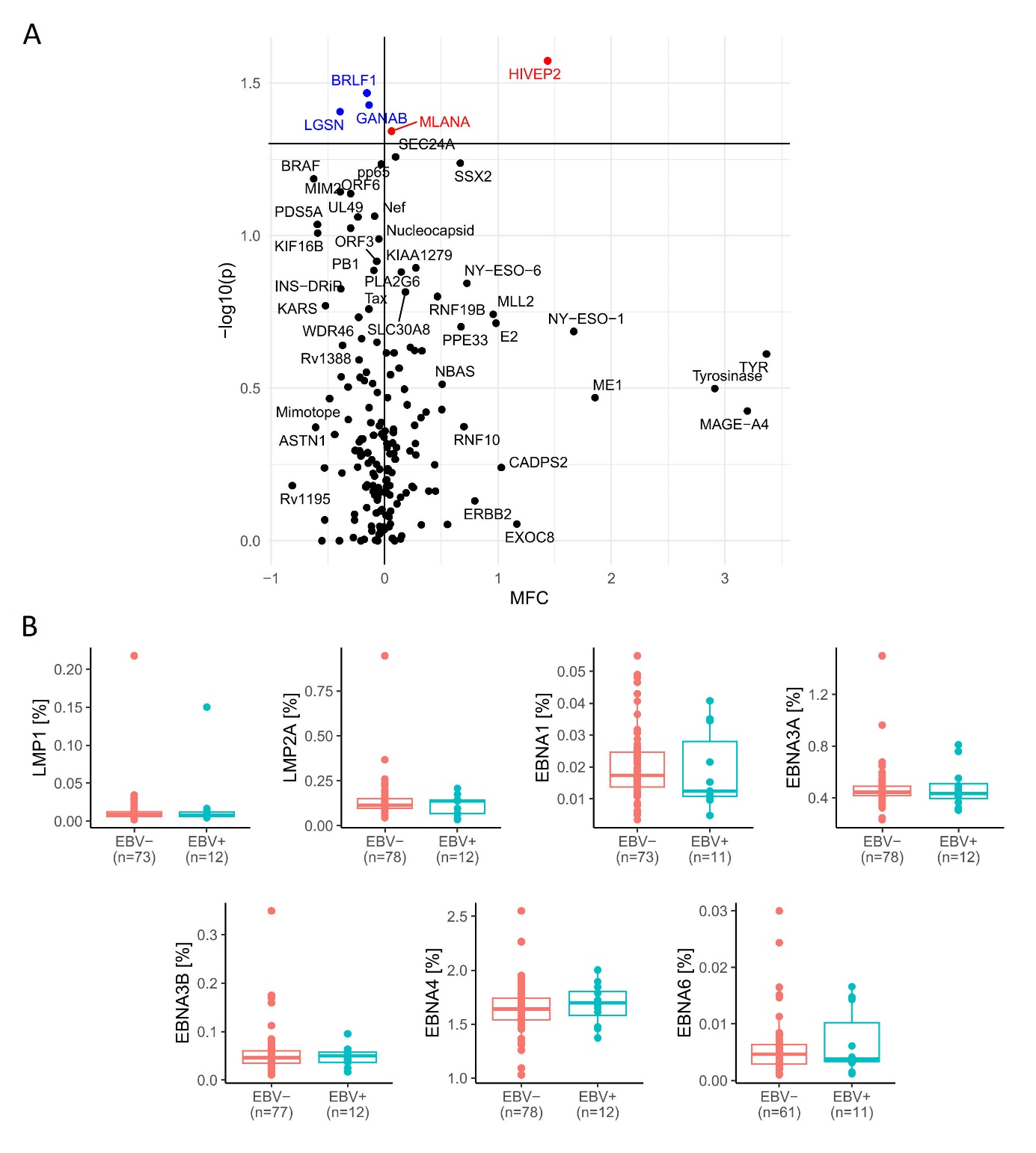


Analysis of T-cell antigen distributions in the tumor microenvironment of treatment-naïve HL in the GHSG phase II NIVAHL trial^11^. Comparison of TCR sequences with known antigen specificity (target antigens, see Supplementary Methods) between EBV- and EBV+ HL patients. Definition of EBV status by LMP1 immunohistochemistry or EBER *in situ* hybridization: EBV- HL = no/low detection of EBV in HRSC; EBV+ HL = detection of EBV in HRSC.

1. Volcanoplot of target antigens compared between EBV- and EBV+ HL. The Mean Fold Change (MFC) indicates the change in the mean frequencies for each target antigen from the EBV- to the EBV+ cohort. Each dot represents one target antigen. P-values were assessed by Wilcoxon-rank sum test. Blue: Antigen frequencies significantly higher in EBV- (p < 0.05); Red: Antigen frequencies significantly higher in EBV+ (p < 0.05).
2. Boxplots of EBV target antigens compared between EBV- and EBV+ HL. Each dot represents the antigen frequency in one patient.

**Supplementary Figure 3**


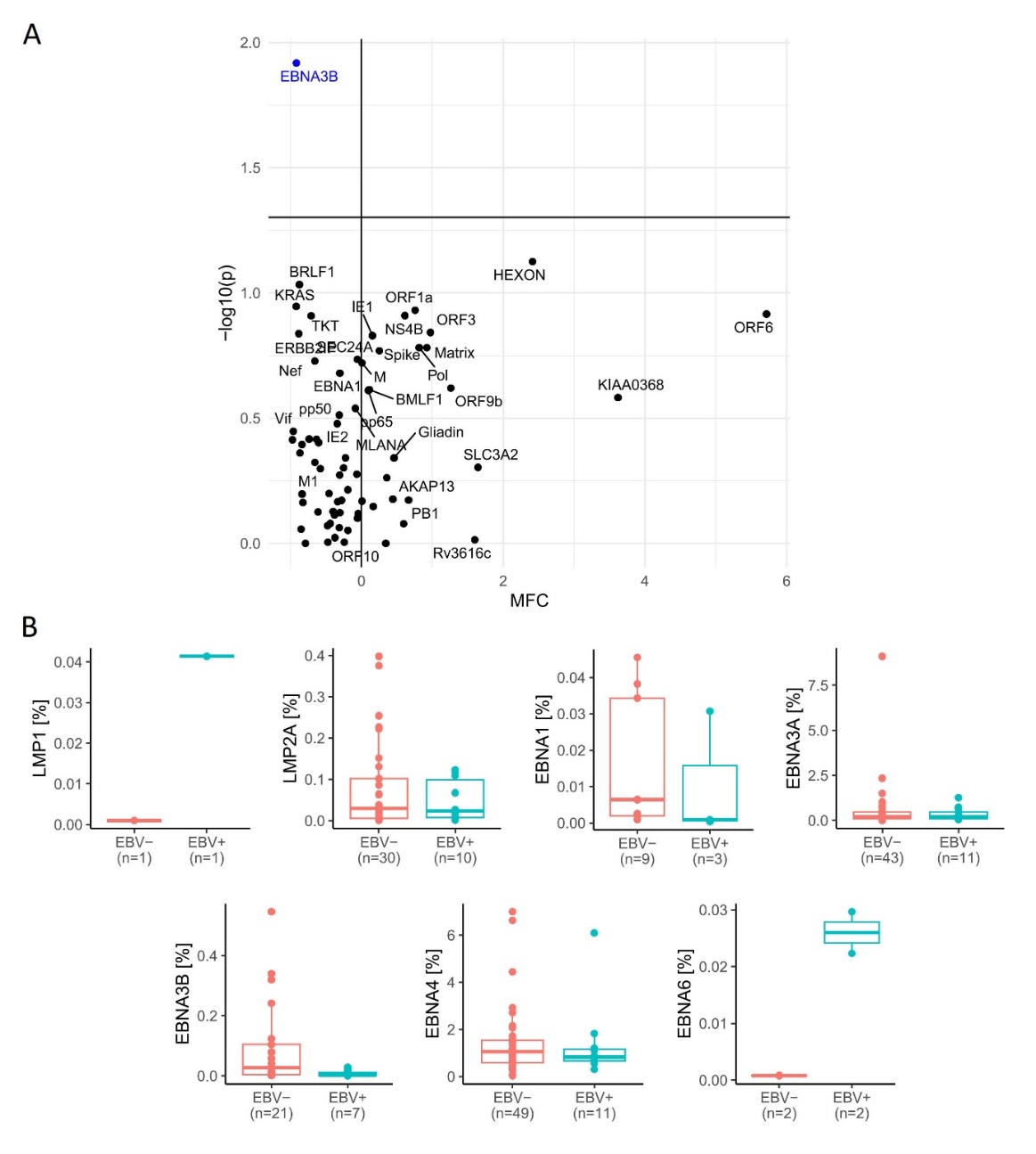


Analysis of T-cell antigen distributions in the tumor microenvironment of treatment-naïve HL in the GHSG phase III HD12^12^ and HD15^13^ trials. Comparison of TCR sequences with known antigen specificity (target antigens, see supplementary methods) between EBV- and EBV+ HL patients. Definition of EBV status by LMP1 immunohistochemistry or EBER *in situ* hybridization: EBV- HL = no/low detection of EBV in HRSC; EBV+ HL = detection of EBV in HRSC.

1. Volcanoplot of target antigens compared between EBV- and EBV+ HL. The Mean Fold Change (MFC) indicates the change in the mean frequencies for each target antigen from the EBV- to the EBV+ cohort. Each dot represents one target antigen. P-values were assessed by Wilcoxon-rank sum test. Blue: Antigen frequencies significantly higher in EBV- (p < 0.05); Red: Antigen frequencies significantly higher in EBV+ (p < 0.05). Please not the low number of cases for LMP1 and EBNA6 both with non-significant results.
2. Boxplots of EBV target antigens compared between EBV- and EBV+ HL. Each dot represents the antigen frequency in one patient.

**Supplementary figure 4**

**
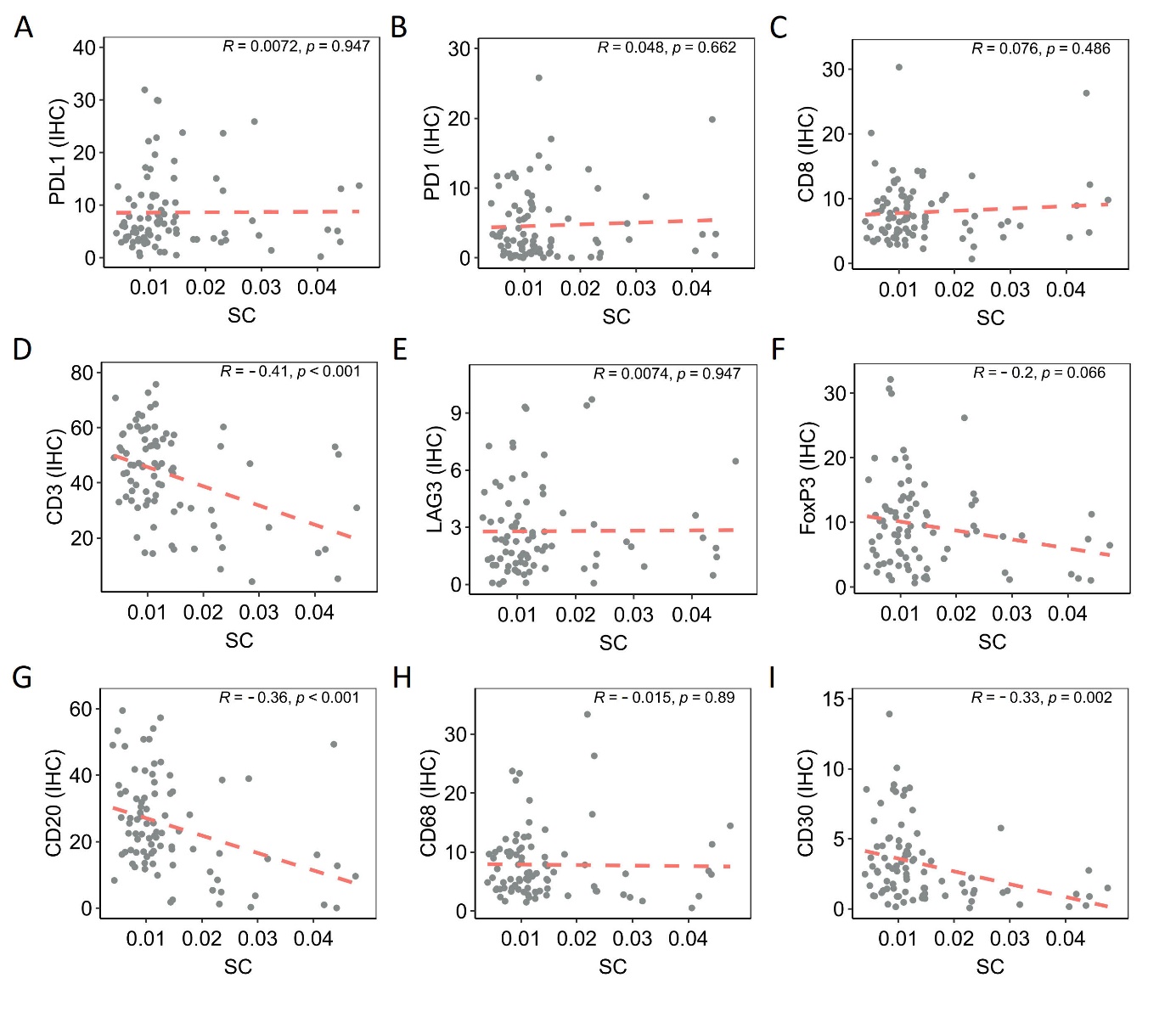
**

Quantitative immunohistochemistry by whole slide image analysis as previously described ^6,14^ and correlation with Simpsons clonality (SC) for PDL1 (A), PD1 (B), CD8 (C), CD3 (D, LAG3 (E), FoxP3 (F), CD20 (G), CD68 (H) and CD30 (I).

**Supplementary Figure 5**


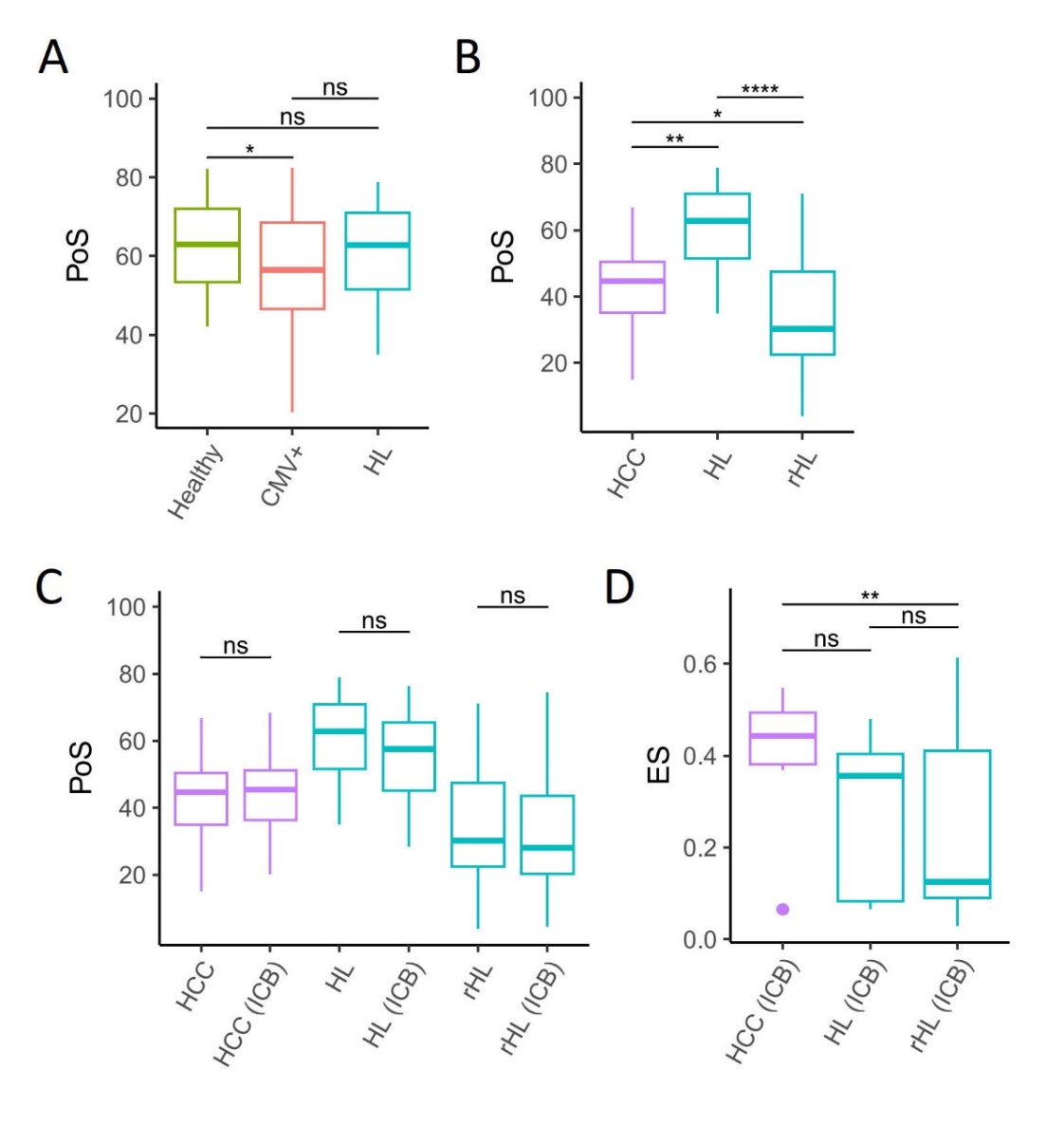


T-cell repertoire analysis in peripheral blood.

1. Percentage of Singletons (PoS) in PBMC of Healthy= healthy donors (n=68), CMV+= CMV infected individuals (n=51), HL= treatment-naive Hodgkin lymphoma specimens (n=21).
2. Percentage of Singletons (PoS) in peripheral blood mononuclear cells (PBMC) of HCC= hepatocellular carcinoma (n=14), HL= treatment-naïve HL (n=21), rHL= relapsed/refractory HL (n=51).
3. Percentage of Singletons (PoS) in peripheral blood mononuclear cells (PBMC) of HCC= paired samples of patients with hepatocellular carcinoma before immune checkpoint blockade (ICB, n=14) and HCC (ICB)= under ICB (n=14), HL= treatment-naïve HL specimens (n=21) and HL (ICB)= subset of HL as paired samples during ICB (at final restaging, n=8), rHL= paired samples of relapsed/refractory HL before ICB (n=51) and rHL (ICB)= during ICB (after 4x Nivolumab, n= 45).
4. Clonal expansion of singletons (ES) in PBMC of HCC, HL and rHL pairs shown in C.

Box and whiskers plots with median indicated as horizontal bar. ns= not significant (p>0.05), * p 0.05-0.01, ** p<0.01, *** p <0.0001, **** p<0.00001

**Supplementary Figure 6**


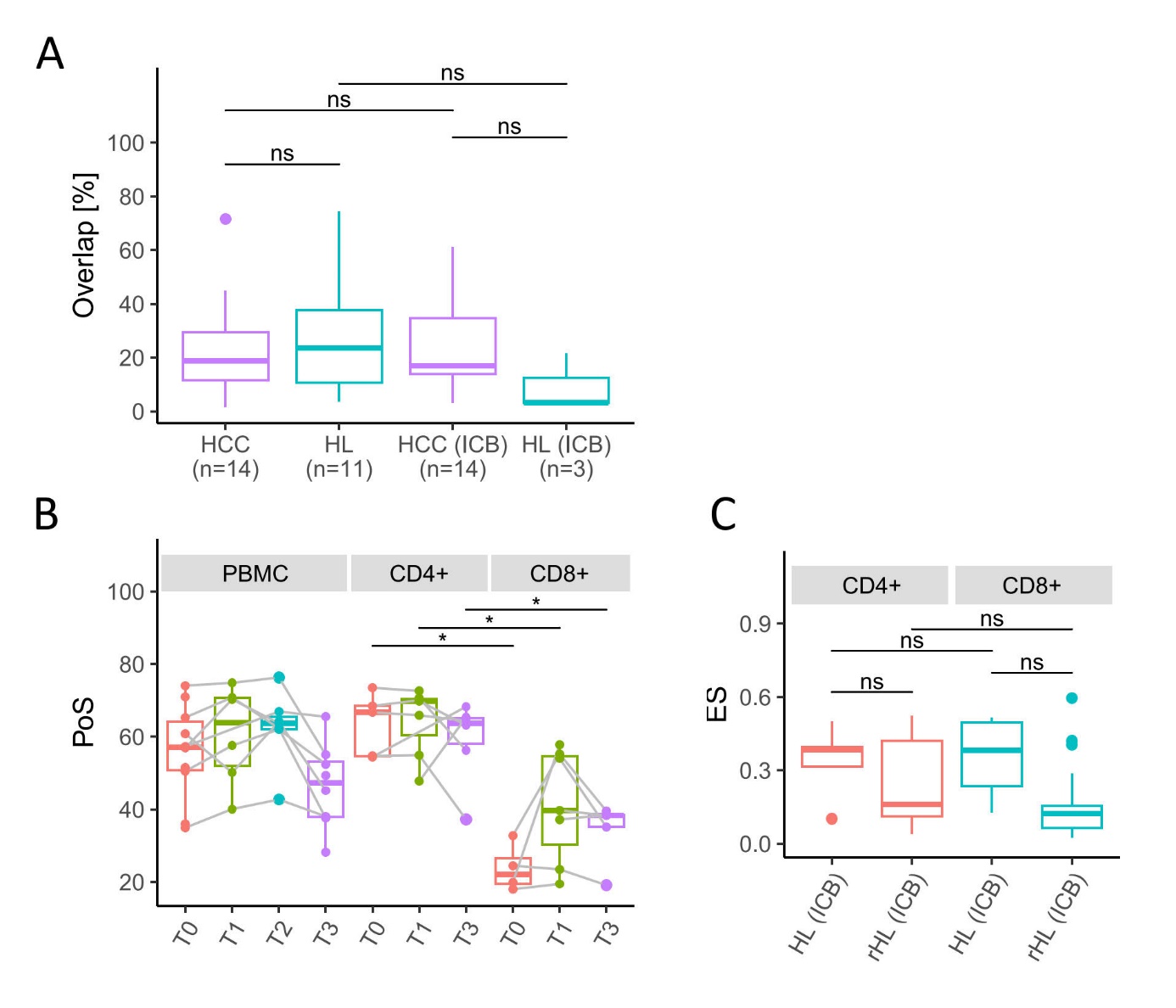


Overlap of TCR sequences in the tumor microenvironment and the peripheral blood and T-cell receptor repertoire analysis of sorted cells in the peripheral blood.

1. Overlap of TCR amino acid sequences in paired samples of tumor microenvironment (TME) and peripheral blood mononuclear cells (PBMC) of treatment naive patients with hepatocellular carcinoma (HCC) and Hodgkin Lymphoma (HL) and of patients during/after immune checkpoint blockade (ICB). Overlap of TCR sequences as percentage of TCR sequences with the same amino acid sequence in the peripheral blood which were also detected in the TME of the same patient.
2. Percentage of Singletons (PoS) peripheral blood mononuclear cells (PBMC) and sorted CD4+ or CD8+ T-cell populations in patients enrolled in the NIVAHL trial ^4^T0= treatment-naïve HL at diagnosis (PBMC n=10, CD4+ n=5, CD8+ n=4), T1= early on-treament, 1-2 weeks after SOT (PBMC n=6, CD4+ n=7, CD8+ n=7), T2= 1st restaging, after 2x Nivolumab-AVD or 4x Nivolumab (PBMC n=8), T3= at final restaging (PBMC n=8, CD4+ n=6, CD8+ n=5).
3. Clonal expansion of singletons (ES) in sorted CD4+ or CD8+ T-cell populations of HL and rHL pairs shown in C.

Box and whiskers plots with median indicated as horizontal bar. ns= not significant (p>0.05), * p 0.05-0.01, ** p<0.01, *** p <0.0001, **** p<0.00001

**Supplementary Figure 7**


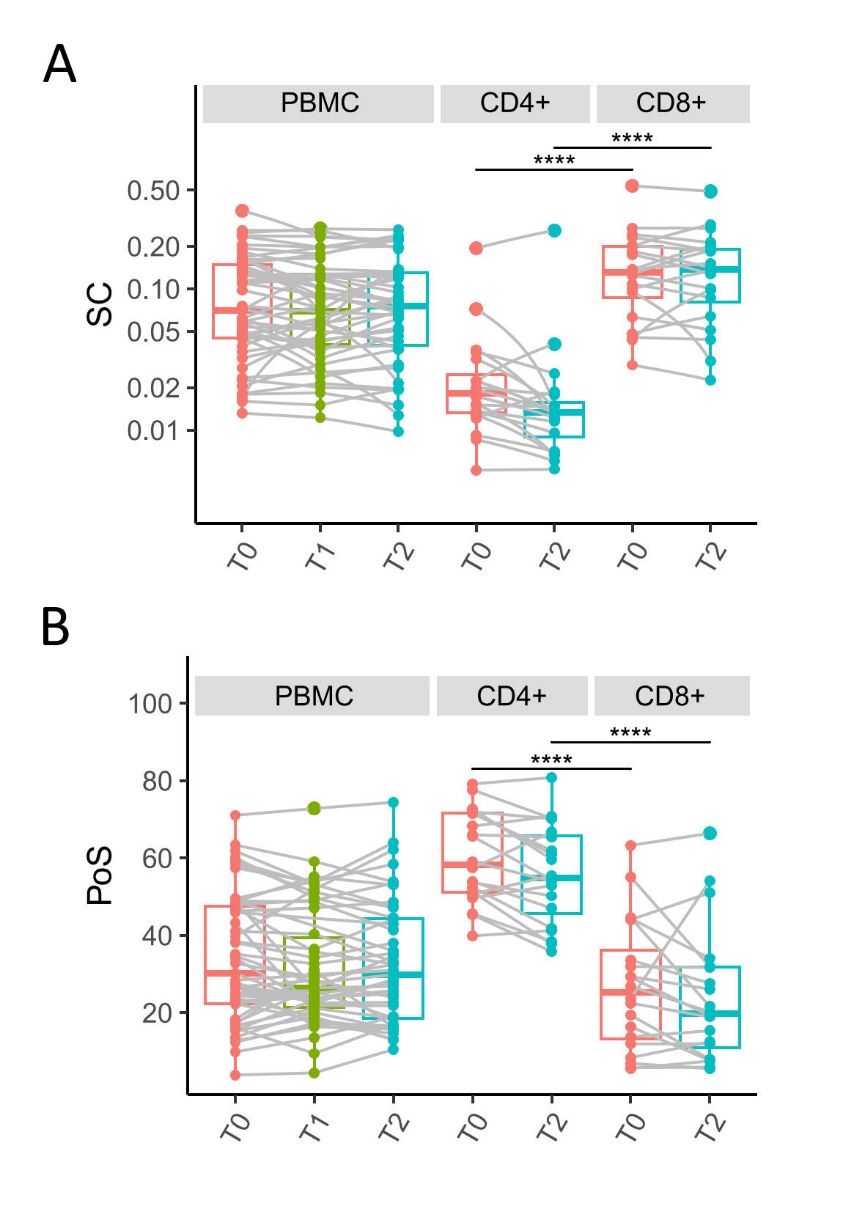


T-cell receptor repertoire analysis of sorted cells in the peripheral blood of patients with relapsed/refractory Hodgkin lymphoma.Simpson’s Clonality (SC, A) and Percentage of Singletons (PoS, B) in peripheral blood mononuclear cells (PBMC) and sorted CD4+ or CD8+ T-cell populations in patients with relapsed/refractory Hodgkin lymphoma (immuneACCESS, [DOI: 10.21417/FZC2020NM](https://doi.org/10.21417/FZC2020NM)^8^). T0= relapsed/refractory Hodgkin lymphoma (PBMC n=51, CD4+ n=20, CD8+ n=20), T1= during second-line ICB (after 2x Nivolumab) (PBMC n=46), T2= during second-line ICB (after 4x Nivolumab) (PBMC n=45, CD4+ n=20, CD8+ n=20).

1. Steinegger M, Söding J. MMseqs2 enables sensitive protein sequence searching for the analysis of massive data sets. *Nat Biotechnol*. 2017;35(11):1026-1028.

2. Shugay M, Bagaev DV, Zvyagin IV, et al. VDJdb: a curated database of T-cell receptor sequences with known antigen specificity. *Nucleic Acids Res*. 2018;46(D1):D419-d427.

3. Yarchoan M, Gane EJ, Marron TU, et al. Personalized neoantigen vaccine and pembrolizumab in advanced hepatocellular carcinoma: a phase 1/2 trial. *Nat Med*. 2024;30(4):1044-1053.

4. Bröckelmann PJ, Goergen H, Keller U, et al. Efficacy of Nivolumab and AVD in Early-Stage Unfavorable Classic Hodgkin Lymphoma: The Randomized Phase 2 German Hodgkin Study Group NIVAHL Trial. *JAMA Oncol*. 2020.

5. Schnitter A, Kohler CW, Reddemann K, et al. Therapeutic targets and microenvironment in sequential biopsies of classical Hodgkin lymphoma at diagnosis and relapse. *Journal of Hematopathology*. 2019;12(1):11-17.

6. Jachimowicz RD, Pieper L, Reinke S, et al. Analysis of the tumor microenvironment by whole-slide image analysis identifies low B cell content as a predictor of adverse outcome in advanced-stage classical Hodgkin lymphoma treated with BEACOPP. *Haematologica*. 2020.

7. Bruggemann M, Kotrova M, Knecht H, et al. Standardized next-generation sequencing of immunoglobulin and T-cell receptor gene recombinations for MRD marker identification in acute lymphoblastic leukaemia; a EuroClonality-NGS validation study. *Leukemia*. 2019;33(9):2241-2253.

8. Bystry V, Reigl T, Krejci A, et al. ARResT/Interrogate: an interactive immunoprofiler for IG/TR NGS data. *Bioinformatics*. 2017;33(3):435-437.

9. Emerson RO, DeWitt WS, Vignali M, et al. Immunosequencing identifies signatures of cytomegalovirus exposure history and HLA-mediated effects on the T cell repertoire. *Nat Genet*. 2017;49(5):659-665.

10. Cader FZ, Hu X, Goh WL, et al. A peripheral immune signature of responsiveness to PD-1 blockade in patients with classical Hodgkin lymphoma. *Nat Med*. 2020;26(9):1468-1479.

11. Bröckelmann PJ, Bühnen I, Meissner J, et al. Nivolumab and Doxorubicin, Vinblastine, and Dacarbazine in Early-Stage Unfavorable Hodgkin Lymphoma: Final Analysis of the Randomized German Hodgkin Study Group Phase II NIVAHL Trial. *J Clin Oncol*. 2023;41(6):1193-1199.

12. von Tresckow B, Kreissl S, Goergen H, et al. Intensive treatment strategies in advanced-stage Hodgkin's lymphoma (HD9 and HD12): analysis of long-term survival in two randomised trials. *Lancet Haematol*. 2018;5(10):e462-e473.

13. Engert A, Plutschow A, Eich HT, et al. Reduced treatment intensity in patients with early-stage Hodgkin's lymphoma. *N Engl J Med*. 2010;363(7):640-652.

14. Grund J, Iben K, Reinke S, et al. Low B-cell content is associated with a CD73-low tumour microenvironment and unfavourable prognosis in classic Hodgkin lymphoma. *Br J Haematol*. 2023.
